# Multisite phosphorylation of P-Rex1 by protein kinase C

**DOI:** 10.18632/oncotarget.12846

**Published:** 2016-10-24

**Authors:** Juan Carlos Montero, Samuel Seoane, Sara García-Alonso, Atanasio Pandiella

**Affiliations:** ^1^ Instituto de Biología Molecular y Celular del Cáncer, CSIC-Universidad de Salamanca, Spain

**Keywords:** P-Rex1, PKC, GEFs, Rac, breast cancer

## Abstract

P-Rex proteins are guanine nucleotide exchange factors (GEFs) that act on the Rho/Rac family of GTP binding proteins. The activity of P-Rex proteins is regulated by several extracellular stimuli. In fact, activation of growth factor receptors has been reported to activate a phosphorylation/dephosphorylation cycle of P-Rex1. Such cycle includes dephosphorylation of serines 313 and 319 which negatively regulate the GEF activity of P-Rex1, together with phosphorylation of serines 605 and 1169 which favour P-Rex1 GEF activity. However, the kinases that regulate phosphorylation at these different regulatory sites are largely unknown. Here we have investigated the potential regulatory action of several kinases on the phosphorylation of P-Rex1 at S^313^, S^319^, S^605^ and S^1169^. We show that activation of protein kinase C (PKC) caused phosphorylation of S^313^, S^319^ and S^1169^. Activation of growth factor receptors induced phosphorylation of S^1169^ through a mechanism that was independent of PKC, indicating that distinct kinases and mechanisms control the phosphorylation of P-Rex1 at different regulatory serines. Genetic and biochemical studies confirmed that the PKC isoform PKCδ was able to directly phosphorylate P-Rex1 at S^313^. Functional studies using cells with very low endogenous P-Rex1 expression, transfected with wild type P-Rex1 or a mutant form in which S^313^ was substituted by alanine, indicated that phosphorylation at that residue negatively regulated P-Rex1 exchange activity. We suggest that control of P-Rex1 activity depends on a highly dynamic interplay among distinct signalling routes and its multisite phosphorylation is controlled by the action of different kinases.

## INTRODUCTION

P-Rex proteins belong to the family of guanine nucleotide exchange factors (GEFs) which act on the Rho/Rac family of GTP binding proteins [1, 2]. In addition, P-Rex proteins may also exert GEF-independent roles, such as the regulation of PTEN function by P-Rex2 [3]. Two different genes, *PREX1* and *PREX2* have been described, each of them coding for three different isoforms [4]. While knockout studies in mice have defined the role of P-Rex1 and P-Rex2 in animal homeostasis [5–8], several other studies have indicated that these proteins may participate in the pathogenesis of certain neoplasias [3, 4, 9–17]. In fact, a link between P-Rex expression and patient outcome has been reported in breast cancer [4, 13]. In melanoma, mutations in P-Rex2 proteins have been described, and preclinical studies demonstrated that ectopic expression of mutated P-Rex2 in melanocytes accelerates tumor formation [11]. Furthermore, when crossed with a murine model of melanoma, *P-Rex1^−/−^* mice are resistant to metastatic spreading of the melanoma cells [10]. In pancreatic cancer, frequent mutations in *PREX2* have also been reported [18].

**Figure 1:**
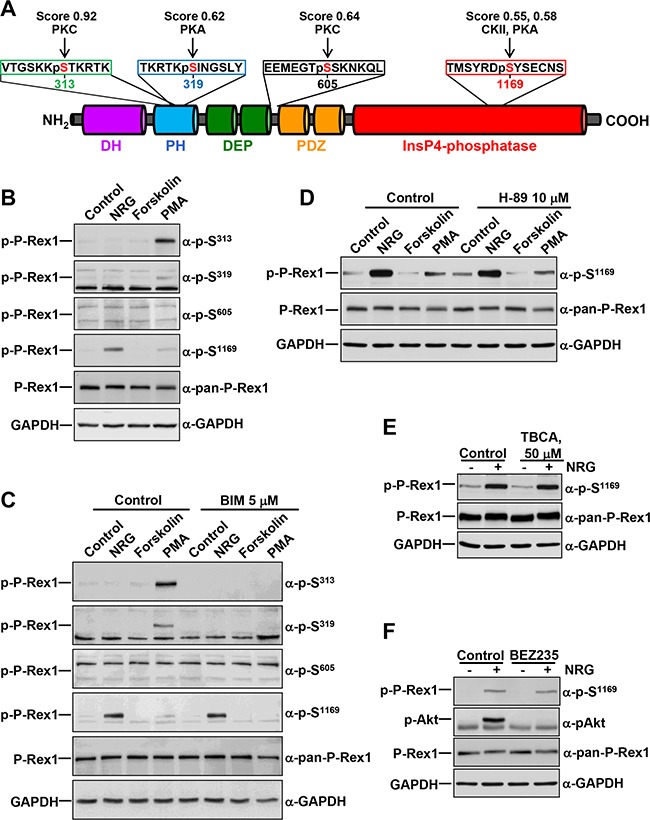
Regulation of P-Rex1 phosphorylation sites by different stimuli **A.** Schematic representation of the different domains of P-Rex1 depicting four regulatory phosphorylation sites, and the peptides recognized by the anti-phospho-P-Rex1 antibodies. The scores and the kinases that could potentially target the different sites recognized by those antibodies are shown. **B.** MCF7 cells were treated with NRG (10 nM), Forskolin (10 μM) or PMA (1 μM) for 15 minutes and lysed. P-Rex1 phosphorylation in serines 313, 319, 605 and 1169 were analyzed by Western blot with anti-phospho-specific antibodies. GAPDH was used as loading control. **C.** MCF7 cells were pretreated with BIM (5 μM) for 1 hour and then treated with NRG, Forskolin or PMA for 15 minutes. Cell lysates were analyzed by Western blot with the indicated antibodies. **D.** MCF7 cells were pretreated with H-89 (10 μM) for 30 minutes and then treated with NRG, Forskolin or PMA for 15 minutes. P-Rex1 phosphorylated at S^1169^ and total P-Rex1 were analyzed by Western blot. GAPDH was used as loading control. **E.** MCF7 cells were pretreated with TBCA (50 μM) for 3 hours and then treated with or without NRG. P-Rex1 phosphorylated at S^1169^ and total P-Rex1 were analyzed as described above. **F.** MCF7 cells were pretreated with BEZ235 (500 nM) for 1 hour and then treated with or without NRG. Cell lysates were analyzed by Western blot with the indicated antibodies. The blots shown come from an experiment that was repeated at least twice.

Several structural domains have been identified in P-Rex proteins [19, 20] (Figure 1A). The N-terminus of these proteins contains an N-terminal Dbl-homology (DH) domain, which is endowed with the typical catalytic domain of the GEFs that act on the Rho/Rac family of GTPases [1, 2]. In addition, P-Rex proteins also include a pleckstrin homology (PH) domain, together with two pairs of DEP and PDZ domains. The C-terminus of P-Rex proteins includes a domain homologous to inositol polyphosphate 4-phosphatase (IP4P). The N-terminal region of P-Rex is absent in isoform 2 of P-Rex1 [4]. On the other side, the C-terminal half of P-Rex2 is absent in isoform P-Rex2b [20, 21].

Phosphorylation of P-Rex represents an important regulatory mechanism. Various reports have demonstrated that P-Rex1 is phosphorylated at multiple sites [4, 22, 23], and such phosphorylations may impact on the GEF function of P-Rex1. In breast cancer cells, stimulation of ErbB receptors or the insulin-like growth factor-1 receptor augments P-Rex1 GEF activity towards Rac by switching on a phosphorylation/dephosphorylation cycle of P-Rex1 [4, 24]. Such cycle includes dephosphorylation of certain residues (Ser^313^ and Ser^319^) accompanied by phosphorylation of other residues (Ser^605^ and Ser^1169^). Mutagenesis as well as functional studies showed that under resting conditions P-Rex1 is phosphorylated at Ser^313^ and Ser^319^ and such phosphorylations inhibit P-Rex1 GEF activity [4]. Of note, Ser^313^ and Ser^319^ are included in the PH domain of P-Rex1, a region whose deletion augments the GEF activity of P-Rex1 in vitro [25]. Therefore, phosphorylation of serines present in the PH domain appears to act as a brake of the GEF activity of P-Rex1. On the other side, phosphorylation of Ser^605^ and particularly Ser^1169^ increases the GEF activity of P-Rex1 [4]. Under resting conditions, phosphorylation of these sites is very low. Upon stimulation of ErbB receptors, phosphorylation at these sites, especially at Ser^1169^ increases, and this facilitates the GEF activity of P-Rex1 towards Rac [4]. Therefore, under resting conditions, P-Rex1 GEF activity is inhibited by phosphorylation of serines located at the PH domain in the N-terminal half of P-Rex1, together with low phosphorylation of serines located at the C-terminal half whose phosphorylation augments P-Rex1 GEF function. Upon stimulation of growth factor receptors, phosphorylation of inhibitory serines decreases, while phosphorylation of stimulatory serines augments, resulting in a net increase in the GEF activity of P-Rex1.

Additional evidence that phosphorylation of P-Rex1 may control its GEF activity has been reported upon activation of β-adrenergic receptors [23]. Agonist-induced activation of these receptors increases the phosphorylation of P-Rex1, likely through protein kinase A (PKA), and such phosphorylation diminishes P-Rex1 GEF activity [23]. Phosphorylation is also expected to affect P-Rex1 intramolecular interaction and modulates binding to subunits Gβγ [26]. Phosphorylation of P-Rex proteins has been reported to inhibit membrane translocation of P-Rex1 in cells exposed to tyrosine kinase inhibitors [27], and may also regulate P-Rex1-dependent ROS production [28]. On the other hand, dephosphorylation of P-Rex1 by the P-Rex1-interacting protein phosphatase 1α [29] increases the GEF activity of P-Rex1 [22]. While a number of phosphorylation sites in P-Rex1 have been identified, and the consequences of such phosphorylation on Rac activity described, the kinases responsible for the direct phosphorylation of P-Rex1 remain largely unidentified. In this report we show that protein kinase C-δ (PKCδ) may act as a P-Rex1 multisite phosphorylating kinase.

## RESULTS

### Bioinformatic estimation of P-Rex1 phosphorylating kinases

In an attempt to identify P-Rex1 phosphorylating kinases, we performed bioinformatic analyses using the NetPhosK 1.0 online tool, which allows predictions of kinase specific eukaryotic protein phosphorylation sites [30]. Upon analysis of a certain uploaded sequence, the online program generates a listing of potentially phosphorylatable sites, the expected phosphorylating kinases, as well as scores (0 to 1). Such scores represent an indication of the likelihood of a kinase potentially targeting a particular site, the higher the score the more likely the site may be phosphorylated by the kinase. Entry of the P-Rex1 sequence generated a listing of 66 serines, 22 threonines and 17 tyrosines which can potentially be phosphorylated by distinct kinases. S^313^, S^319^, S^605^ and S^1169^ were included among those sites and we centered our attention on those residues because of their reported regulatory role on P-Rex1 GEF activity [4]. These studies predicted PKC to be able to phosphorylate S^313^ (score: 0.92) and S^605^ (score: 0.64) of P-Rex1 (Figure 1A). In addition, PKA was expected to be able to phosphorylate S^319^ (score: 0.62) and S^1169^ (score: 0.58). The latter residue was also expected to be a target for casein kinase II (CKII) (score: 0.55).

To further explore P-Rex1 phosphorylating kinases in silico, we also used the Scansite 3 beta online tool. Scansite searches for specific motifs within proteins that may be phosphorylated by mammalian protein kinases or bind to domains such as SH2 domains, 14-3-3 domains or PDZ domains [31]. A total of 70 kinase/interaction motifs are analyzed by this program. S^313^ was identified by the program as a target for kinases of the PKC family (Supplementary Table S1). In contrast, high stringency analysis failed to identify S^319^, S^605^ and S^1169^ as potential phosphorylation sites of any of the kinases present in the Scansite database. Of note, S^313^ offered the highest surface availability (6.943) as compared to other predicted phosphorylation sites whose surface availability was below 3.

### P-Rex1 is phosphorylated by kinases activated through different signaling pathways

The in silico studies pointed to PKC and PKA as potential kinases which may control the phosphorylation of the regulatory S^313^, S^319^, S^605^ and S^1169^ sites. This, together with previously published data indicating that PKA activity may phosphorylate P-Rex1 [23] led us to explore the effect of these kinases on the phosphorylation of S^313^, S^319^, S^605^ and S^1169^. To this end, MCF7 breast cancer cells were treated with the PKC agonist Phorbol 12-myristate,13-acetate (PMA), or with forskolin, which is known to stimulate PKA activity by augmenting cAMP levels. These cells, which mainly express isoform 1 of P-Rex1, were treated with these drugs for 15 minutes and the phosphorylation of S^313^, S^319^, S^605^ or S^1169^ was analyzed by Western blotting using antibodies which specifically recognize those residues when phosphorylated [4]. The sequences in P-Rex1 against which these antibodies were raised are illustrated in Figure 1A. Treatment with PMA caused a strong up-regulation of the phosphorylation of P-Rex1 at S^313^ (Figure 1B). In addition, PMA also increased phosphorylation of P-Rex1 at S^319^ and S^1169^, although to a lesser extent than in the case of S^313^ (Figure 1B). In contrast, no effect on the phosphorylation of P-Rex1 at any of the four analyzed residues was observed in cells treated with forskolin. Treatment with dibutyryl-cAMP (dbcAMP), which is expected to directly activate PKA also failed to affect phosphorylation of any of the four analyzed residues (data not shown).

As formerly reported [4] Neuregulin (NRG), which binds to ErbB3 and activates ErbB2 and ErbB3 receptors in MCF7 cells, increased S^1169^ phosphorylation in these cells. Moreover, activation of NRG receptors was the strongest stimulus found to up-regulate S^1169^ phosphorylation (Figure 1B). Phosphorylation of S^605^ was not up-regulated by PKC or PKA agonists, suggesting that phosphorylation of this residue must rely on kinases other than PKC or PKA.

The variability of sites and conditions which regulated P-Rex1 phosphorylation at the distinct residues suggested that several kinases/signaling pathways could be implicated in the control of such phosphorylations. To investigate this possibility, the action of different compounds expected to activate/inhibit P-Rex1 phosphorylation at S^313^, S^319^, S^605^ or S^1169^ was studied. The PKC inhibitory compound BIM fully prevented PMA-induced phosphorylation of P-Rex1 at S^313^ and S^319^ (Figure 1C). BIM also affected PMA-induced phosphorylation of S^1169^, although in this case the inhibition was less easily evidenced due to the weak stimulatory effect of PMA on the phosphorylation of that site. BIM was unable to affect NRG-induced phosphorylation of P-Rex1 at S^1169^, indicating that kinases other than PKC isozymes should be responsible for the action of NRG on S^1169^ phosphorylation.

Since PKA and CKII were identified by the NetPhosK online tool as S^1169^ phosphorylating candidates, the possibility that those kinases could act as S^1169^ phosphorylating kinases upon activation of the ErbB receptors by NRG was explored. Phosphorylation of S^1169^ by NRG was insensitive to the PKA antagonist H-89 (Figure 1D), as well as to the CKII antagonist TBCA (Figure 1E), suggesting that the NRG-activated kinase responsible for such phosphorylation was unrelated to those kinases. In addition, the lack of inhibition of PMA-induced S^1169^ phosphorylation by H-89 suggests that PKA does not participate in PKC-induced phosphorylation of that site. It is also worth noting that forskolin caused a small decrease in S^1169^ phosphorylation (Figure 1B and 1C), suggesting that PKA activity may inhibit S^1169^ phosphorylation, rather than acting as a kinase which promotes such phosphorylation.

P-Rex1 was initially identified as a phosphatidylinositol-3,4,5-P3 (PI3K)-regulated GEF [19]. Because of this, and the fact that NRGs are strong activators of this pathway in MCF7 cells, the possibility that the PI3K route could mediate the stimulation of S^1169^ phosphorylation by NRG was explored. Preincubation of MCF7 cells with BEZ235, a dual PI3K/mTOR inhibitor [32] also failed to affect NRG-induced phosphorylation of S^1169^ (Figure 1F). As expected, NRG provoked strong up-regulation of Akt phosphorylation at S^473^ (Figure 1F) and S6 (data not shown) which are used as readouts of the activity of the PI3K/mTOR pathway. BEZ235 fully prevented NRG-induced phosphorylation of Akt at S^473^ (Figure 1F) and S6 (data not shown), suggesting that NRG-induced phosphorylation of P-Rex1 at S^1169^ was not linked to the PI3K/mTOR pathway.

Taken together, the above data suggested that P-Rex1 phosphorylation may be controlled by different pathways, and may be caused by PKC-dependent as well as PKC-independent mechanisms.

### Kinetics of PKC-induced P-Rex1 phosphorylation

**Figure 2:**
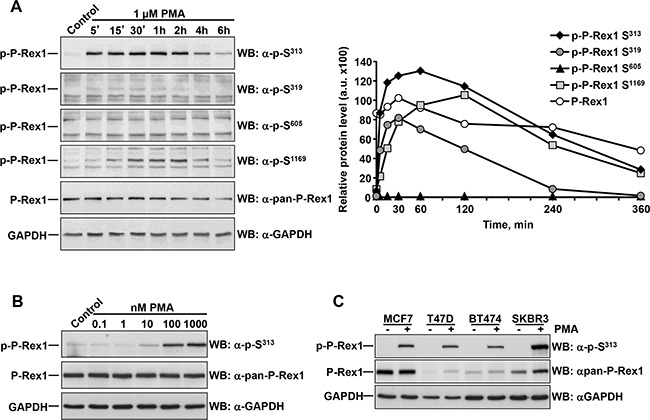
Dose-response and time-course of the effect of PMA on P-Rex1 phosphorylation **A.** MCF7 cells were treated with PMA for the times indicated and lysed. P-Rex1 phosphorylated at S^313^, S^319^, S^605^, S^1169^, total P-Rex1 and GAPDH expression were analyzed by Western blot, and the levels of phosphorylated and total P-Rex1 quantitated and plotted. The graph represents the relative protein levels of total and phosphorylated P-Rex1 with respect to the GAPDH levels. **B.** MCF7 cells treated with the indicated doses of PMA were lysed and the levels of P-Rex1 pS^313^ and total P-Rex1 analyzed by Western. **C.** MCF7, T47D, BT474 and SKBR3 cells were treated with PMA for 15 minutes and lysed. P-Rex1 pS^313^, total P-Rex1 and GAPDH expression were analyzed by Western blot. The blots shown come from an experiment that was repeated at least twice.

Time-course (Figure 2A) analyses confirmed the stimulation of P-Rex1 S^313^, S^319^ and S^1169^ phosphorylations upon activation of PKC by PMA. Such phosphorylations were rapid, being detected at the first time point analyzed (5 minutes), and sustained for several hours (Figure 2A). Of note, maximum S^1169^ phosphorylation after PMA treatment reached a peak at times delayed with respect to the phosphorylation of P-Rex1 at S^313^. Phosphorylation of P-Rex1 at S^319^ peaked before that of S^1169^. Dose response studies indicated that PMA caused maximal stimulation of phosphorylation of P-Rex1 at S^313^ at concentration 100 nM and above (Figure 2B).

We explored whether the effect of PMA on S^313^ P-Rex1 phosphorylation was a general cell biological phenomenon, and not particular of MCF7 cells. For this purpose, three additional breast cancer cell lines, in addition to MCF7 cells, were treated with PMA and P-Rex1 phosphorylation at S^313^ measured. As shown in Figure 2C, stimulation of the different cell lines with PMA resulted in up-regulation of S^313^ in all of them, even though their total P-Rex1 levels varied.

### PKCδ knockdown reduces PMA-stimulated S^313^ phosphorylation

**Figure 3:**
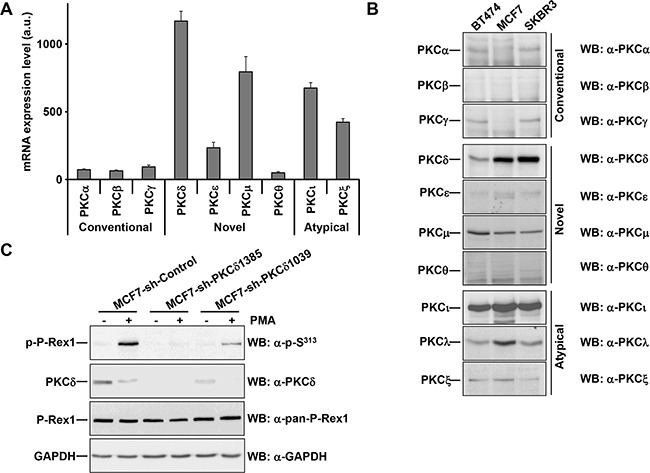
PKCδ knockdown reduces the phosphorylation of PMA-stimulated P-Rex1 pS^313^ **A.** Expression of the major atypical, conventional and novel PKC isoforms in MCF7 cells using data generated from expression arrays. The graph represents the levels of expression (in arbitrary units, mean±s.d., n=3) of different isoforms of PKC in MCF7 cells. **B.** BT474, MCF7 and SKBR3 cells were lysed. Expression of the major atypical, conventional and novel PKC isoforms were analyzed by Western blot with the antibodies indicated. **C.** MCF7 cells infected with the short hairpins of PKCδ (sequences #1385 and #1039) or a short hairpin control were treated with and without PMA for 15 minutes and lysed. The levels of P-Rex1 pS^313^, PKCδ, P-Rex1 and GAPDH were analyzed by Western blot. The blots shown come from an experiment that was repeated at least twice.

To gain additional insights into the mechanism involved for the PKC-induced phosphorylation of P-Rex1 in S^313^, we attempted to identify the isoform of that kinase responsible for such phosphorylation. We first analyzed expression of the major atypical, conventional and novel PKC isoforms in MCF7 cells using data from expression arrays [33]. These studies indicated that PKCδ was the most abundant PKC isoform, followed by μ, ι, and ζ (Figure 3A). Western blotting analyses corroborated the high expression of PKCδ and PKCι in MCF7 cells (Figure 3B). SKBR3 cells, in which a strong induction of S^313^ phosphorylation was observed upon stimulation of PKC activity by PMA (Figure 2C) also showed high levels of PKCδ and PKCι expression (Figure 3B). Lower levels of PKCδ were observed in BT474 cells on which treatment with PMA also resulted in S^313^ phosphorylation.

The coincidence of the high array expression data and the high protein levels of PKCδ as detected in the Western blotting experiments pointed to this PKC isoform as the principal candidate to mediate P-Rex1 phosphorylation upon PMA treatment. To analyze to which extent PKCδ could act as an intermediate in the phosphorylation of P-Rex1 at S^313^, knockdown experiments were performed. MCF7 cells were infected with five lentiviral particles which included five different PKCδ-interfering sequences and the two of them which caused better degrees of interference selected. MCF7 cells infected with lentivirus containing either of the two sequences (#1385 and #1039) showed a substantial reduction in PKCδ levels (Figure 3C). In the PKCδ knocked-down cells, phosphorylation of P-Rex1 at S^313^ upon treatment with PMA was strongly reduced, especially in cells infected with the more efficient #1385 lentiviruses. Of note, treatment with PMA decreased the levels of PKCδ. These knockdown experiments indicated that PKCδ had to be present to mediate the effect of the phorbol ester on the phosphorylation of P-Rex1 at S^313^.

### Direct phosphorylation of P-Rex1 at S^313^ by PKCδ

**Figure 4:**
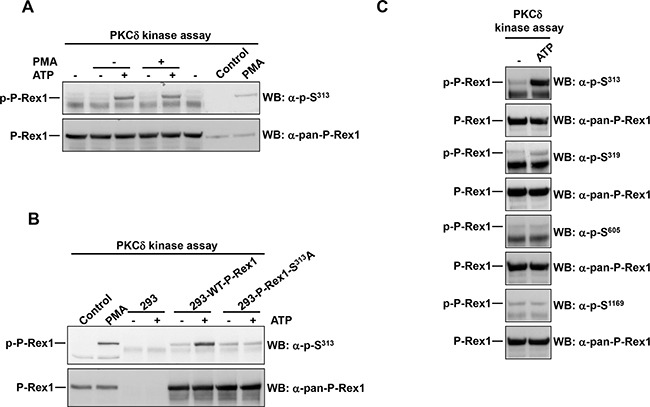
PKCδ phosphorylates P-Rex1 at Ser^313^ **A.** P-Rex1 immunoprecipitated from extracts of MCF7 cells was used as substrate in a PKCδ in vitro kinase assay. ATP or PMA were added in the kinase assay where indicated. After completion of the kinase assay, P-Rex1 pS^313^ and total P-Rex1 levels were analyzed by Western blot. **B.** P-Rex1 was immunoprecipitated from extracts of 293 cells transfected with wild type P-Rex1 or P-Rex1 mutated in serine 313 to alanine and used as substrate in PKCδ in the in vitro kinase assay. The levels of P-Rex1 pS^313^ and total P-Rex1 were analyzed by Western blot. **C.** MCF7 cells were lysed and the extracts were used to immunoprecipitate P-Rex1. Then the PKCδ kinase assay was performed. P-Rex1 phosphorylation levels in serine 313, 319, 605, 1169 and total P-Rex1 were analyzed by Western blot with anti-phospho-specific and anti-pan-P-Rex1 antibodies. The blots shown come from an experiment that was repeated at least twice.

The possibility that P-Rex1 S^313^ could be directly phosphorylated by PKCδ was explored. To this end, P-Rex1 was immunoprecipitated from extracts of MCF7 cells and used as substrate in a PKCδ in vitro kinase assay. After the kinase assays were terminated, reaction mixtures were run in SDS-PAGE gels and blots were probed with the anti-pS^313^ antibody. As shown in Figure 4A, in the absence of ATP no phosphorylation of P-Rex1 was observed. In contrast, addition of ATP to the in vitro kinase reaction resulted in phosphorylation of P-Rex1 at S^313^. Such phosphorylation was unaffected by the in vitro incubation of the reaction mixtures with PMA, indicating that under these in vitro conditions addition of the phorbol ester is not required for the stimulation of P-Rex1 S^313^ phosphorylation.

To verify that the direct phosphorylation of P-Rex1 occurred at S^313^ and that was detected specifically by the anti-pS^313^ antibody, 293 cells which express low amounts of autochthonous P-Rex1, were transfected with a plasmid coding either wild type P-Rex1 or a mutant form of P-Rex1 in which S^313^ was mutated to alanine. Wild type P-Rex1 and P-Rex1-S^313^A were immunoprecipitated from these cells, and used as substrates in the in vitro kinase assay. In the presence of ATP, only the wild type transfected form was able to be phosphorylated by PKCδ in the in vitro kinase assay conditions (Figure 4B).

To analyze whether PKCδ could in vitro phosphorylate other P-Rex1 residues, in vitro kinase assays were performed and the blots probed with antibodies to S^319^, S^605^ and S^1169^. As shown in Figure 4C, a weak phosphorylation signal was obtained in the anti-pS^319^ blot. This finding was in line with the data obtained in vivo upon activation of PKC by treatment of the cells with PMA (Figure 1B). In contrast, no phosphorylation of S^605^ and S^1169^ was detected, indicating that PKCδ cannot directly phosphorylate these residues in vitro. Taken together, these results indicate that PKCδ may act as a direct P-Rex1 S^313^ and S^319^ kinase. In contrast, S^1169^ could not be directly phosphorylated by PKCδ in vitro. This is relevant, since PKC activation was able to increase phosphorylation of S^1169^ in vivo (Figure 1B).

### Phosphorylation of P-Rex1 at S^313^ restricts its Rac-GEF activity

**Figure 5:**
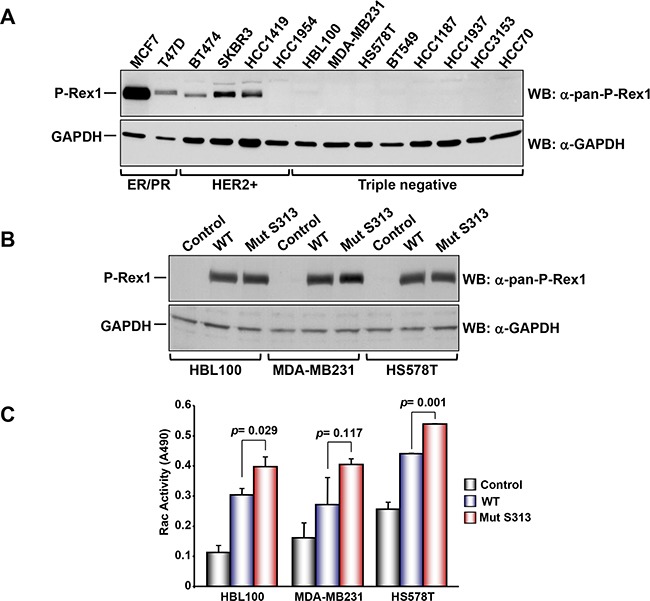
**A.** Analysis of the expression of P-Rex1 in different breast cancer cell lines. The different cells lines were lysed and the expression of P-Rex1 was analyzed by Western blot. **B.** HBL100, MDA-MD231 and HS578T cells were transfected with vectors coding for wild type P-Rex1 or P-Rex1 mutated in S^313^A and lysed. The expression of the different forms was analyzed by Western blot with the anti-pan-P-Rex1 antibody. GAPDH was used as loading control. **C.** Rac activity in the cell lines described above and transfected with wild type P-Rex1 or P-Rex1 mutated in S^313^A. The blots shown come from an experiment that was repeated at least twice.

To explore the functional consequences of P-Rex1 S^313^ phosphorylation on its Rac GEF activity we analyzed several breast cancer cell lines for their expression of P-Rex1, with the aim of encountering a cell line with low/undetectable levels of P-Rex1 in which we could analyze the effect of expression of P-Rex1 or P-Rex1-S^313^A on Rac activity without interference from endogenous P-Rex1. Cell lines belonging to the estrogen receptor/progesterone receptor subtype (MCF7 and T47D) as well as cell lines of the HER2+ subtype (BT474, SKBR3, HCC1419) expressed P-Rex1, while HCC1954 which also belongs to the HER2+ subtype did not express P-Rex1 (Figure 5A). Interestingly, all the triple negative subtype cell lines (HBL100, MDA-MB231, HS578T, BT549, HCC1187, HCC1937, HCC3153 and HCC70) did not express P-Rex1. Because of this characteristic, wild type or S^313^A mutant P-Rex1 were transfected into three of these triple negative cells, and the effect on Rac activity was analyzed. Western blotting analyses failed to detect any P-Rex1 in cells transfected with the empty vector (Figure 5B). In contrast, exogenous P-Rex1, either wild type, or the S^313^A mutant were readily detected, and their amounts were similar. Resting Rac activity was variable among the distinct cell lines studied (Figure 5C). Expression of wild type P-Rex1 significantly augmented resting Rac activity in all the three cell lines. Moreover, expression of the mutant P-Rex1 form had a higher stimulatory effect on Rac activity than the wild type-expressed protein. Together, these data indicate that the levels of P-Rex1 as well as its phosphorylation status are important regulators of Rac activity.

## DISCUSSION

We initiated this work with the purpose of identifying kinases responsible for controlling P-Rex1 phosphorylation. Previous studies demonstrated that P-Rex proteins are phosphorylated at different residues, including serines at positions 313, 319, 605 and 1169. In breast cancer cells, activation of growth factor receptors was shown to modify the phosphorylation status of those four P-Rex1 sites, and such phosphorylations were linked to changes in the GEF activity of P-Rex1 [4, 24]. Moreover, the activation of growth factor receptors triggered a phosphorylation/dephosphorylation cycle which caused dephosphorylation of S^313^ and S^319^, as well as phosphorylation of S^605^ and S^1169^. Mutagenesis and functional studies demonstrated that phosphorylation of S^313^ and S^319^ inhibits P-Rex1 GEF activity, and activation of growth factor receptors resulted in dephosphorylation of these residues. Such dephosphorylations should result from augmentation of the activity of phosphatases, a decrease in P-Rex1 phosphorylating kinases, or a combination of both situations. On the other side, activation of growth factor receptors provoked phosphorylation of residues S^605^ and S^1169^. Similar mutagenesis/functional studies demonstrated that such phosphorylations favored the GEF activity of P-Rex1 towards Rac. The net result of the switching on of such phosphorylation/dephosphorylation cycle is an increase in Rac activity upon stimulation of growth factor receptors, particularly those endowed with tyrosine kinase activity. Another more recent report described additional phosphorylation sites and their regulation by protein phosphatase 1α [22]. While the above commented reports indicate that P-Rex1 phosphorylation is highly dynamic, the mechanisms and kinases that control the phosphorylation status of these sites remain largely uncharacterized.

Bioinformatic analyses pointed to PKC, CKII and PKA as potential P-Rex1 kinases. Biochemical studies indicated that PKC activation caused phosphorylation of P-Rex1 at several residues, particularly S^313^. In contrast, PKA activation using forskolin or the cAMP analog dbcAMP were unable to augment P-Rex1 phosphorylation at any of the residues analyzed. Moreover, treatment with the PKA inhibitor H-89 failed to inhibit P-Rex1 phosphorylation at S^1169^. Since PKA has been reported to phosphorylate P-Rex1 and such phosphorylations inhibit the PIP3- and Gβγ-stimulated P-Rex1 guanine nucleotide exchange activity on Rac [23], it is likely that the sites phosphorylated by PKA do not correspond to the ones analyzed in this work.

Phosphorylation of P-Rex1 at S^313^, S^319^ and S^1169^ upon stimulation with PKC agonists could be completely prevented by the wide spectrum PKC inhibitor BIM. However, NRG induced phosphorylation of P-Rex1 at S^1169^ was insensitive to BIM, indicating that kinases other than PKC are involved in the phosphorylation of P-Rex1 at that residue upon activation of ErbB receptor tyrosine kinases. Growth factor-induced phosphorylation of P-Rex1 at S^1169^ was insensitive to all the inhibitors tested, including agents that inhibit PKA, CKII, PI3K/mTOR. Given the role of the phosphorylation of that residue in the regulation of the GEF activity of P-Rex1, it will be interesting to decipher the kinase(s) involved in the regulation of such phosphorylation upon activation of growth factor receptors. Since the inhibitor studies failed to define the S^1169^ kinase, the use of alternative approaches, e. g. kinome targeting with siRNA libraries, may help in the identification of such kinase. These results were taken to indicate the highly regulated nature of P-Rex1 phosphorylation, and the fact that distinct kinases/signaling routes control multisite phosphorylation of P-Rex1.

Genetic studies, which included gene expression profiling as well as knockdown experiments identified PKCδ as an important kinase in the regulation of P-Rex1 phosphorylation at S^313^. Moreover, in vitro kinase experiments confirmed that this kinase could directly phosphorylate P-Rex1 at S^313^. However, our studies cannot exclude that kinases other than PKCδ may phosphorylate P-Rex1 at S^313^. While in vivo activation of PKC resulted in phosphorylation of P-Rex1 at S^1169^, the in vitro studies failed to demonstrate direct PKCδ phosphorylation of that residue. It is therefore possible that such failure may be caused by the requirement of another component absent in the in vitro kinase assay. Another possibility is that a PKC isoform other than PKCδ may be involved in the phosphorylation of S^1169^.

Crystallographic analyses indicated that Rac interacts with the DH domain of P-Rex [34]. Residues S^313^ and S^319^ of P-Rex1 are located within the β3-β4 loop of the PH domain of P-Rex1, a region that is not expected to directly interact with Rac. However, those studies have shown that the β3-β4 loop is endowed with a high degree of mobility and surface availability. This property may facilitate accessibility for interaction with other regulatory regions of P-Rex1 or with other proteins, such as kinases or phosphatases that may regulate the GEF action of P-Rex1. The high surface availability of S^313^ could favor its phosphorylation, causing a conformational change that could facilitate phosphorylation of other P-Rex sites. In fact, the delayed time course of phosphorylation of other residues with respect to S^313^ could be due to such phenomenon, or could be indirect. Phosphorylation of P-Rex1 at S^313^ has been formerly reported to negatively regulate the GEF function of P-Rex1 against Rac [4]. Those studies were performed on MCF7 cells firstly depleted of endogenous P-Rex1 and then reconstituted with either wild type or a mutant form of P-Rex1 in which S^313^ was substituted by non-phosphorylatable alanine. While informative, those studies had limitations, since the knockdown of P-Rex1 was not complete, and some endogenous protein remained. To better assess the relevance of the phosphorylation of P-Rex1 at S^313^ we analyzed several breast cancer cell lines which belong to the three major clinicopathological subtypes. These studies informed that expression of P-Rex1 was very low in the cell lines representative of the triple negative subgroup. We took advantage of the lack of substantial expression of P-Rex1 in these cell lines to explore the relevance of P-Rex1 phosphorylation at S^313^ on Rac activity. When expressed at similar levels, the mutated form of P-Rex1 which contained a non phosphorylatable residue at position 313 had a stronger effect on Rac activity than wild type P-Rex1. This is consistent with the concept that phosphorylation of P-Rex1 at S^313^ restricts P-Rex1 GEF activity towards Rac. We observed that expression of wild type P-Rex1 form in the three cell lines tested substantially augmented resting Rac activity, indicating that overexpression of P-Rex by itself is sufficient to raise Rac activity. This conclusion is relevant, since elevated Rac activity may contribute to progression of diseases such as cancer. Moreover, the expression of elevated levels of P-Rex1 has been linked to a worse patient outcome in breast cancer, particularly in the luminal type [4, 13]. A mechanism that may link P-Rex proteins to breast cancer outcome is their role in the regulation of metastatic dissemination. In fact, a recent report [35] suggested that P-Rex may control a genetic program that facilitates metastatic dissemination. Interestingly, in triple negative breast cancer cell lines P-Rex expression was very low, even though such subtype of breast tumor is highly metastatic. It is possible that in this triple negative tumors other GEFs may substitute P-Rex activity. In depth exploration of the role of P-Rex in triple negative breast cancer has not been carried out, but should be made to better understand the relevance of P-Rex in the breast cancer panorama. The relevant role of P-Rex in breast cancer, together with the presence of mutated forms of P-Rex in melanomas [11] indicates that P-Rex proteins may be considered therapeutic targets, and gives support to the development of additional studies aimed at elucidating the role and regulation of P-Rex1 function.

## MATERIALS AND METHODS

### Reagents and antibodies

Cell culture media, puromycin, PMA [36], dbcAMP [37] and Forskolin [38] were purchased from Sigma-Aldrich (St Louis, MO, USA). Fetal bovine serum (FBS) and penicillin/streptomycin were from Invitrogen (Gaithersburg, MD, USA). Protein A-Sepharose was from GE Healthcare Life Sciences (Piscataway, NJ, USA). TBCA [39] and H-89 [40] were from Santa Cruz Biotechnology (Santa Cruz, CA, USA). BIM [36] was from Calbiochem (La Jolla, CA, USA). BEZ235 was from LC Laboratories (Woburn, MA, USA). Neuregulin (NRG) was from Prospec (Rehovot, Israel). Other generic chemicals were purchased from Sigma, Roche Biochemicals or Merck (Darmstadt, Germany).

The antibody against GAPDH was purchased from Santa Cruz Biotechnology. The anti-PKC antibodies were from BD Transduction Laboratories, Inc (San Jose, CA, USA). The anti-pAKT (S^473^) antibody was from BD Biosciences. The antibodies against P-Rex1 (anti-pan-P-Rex) and phospho P-Rex1 (anti-pS^313^, anti-pS^319^, anti-pS^605^ and anti-pS^1169^) have been described [4]. Horseradish peroxidase conjugates of anti-rabbit and anti-mouse immunoglobulin G were from Bio-Rad Laboratories (Hercules, CA, USA).

### Cell culture, infection with lentivirus and transfections

All cell lines were cultured at 37°C in a humidified atmosphere in the presence of 5% CO_2_ and 95% air. Cells were grown in DMEM or in RPMI medium containing high glucose concentration (4,500 mg/liter) and antibiotics (penicillin at 100 mU/ml, streptomycin at 100 μg/ml) and supplemented with 10% FBS. Cell lines were provided by Drs. J. Losada and A. Balmain (originally from Dr. J. W. Gray's Laboratory who in turn obtained them from the ATCC or from collections developed in the laboratories of Drs. S. Ethier and A. Gazdar, to avoid errors occurring when obtained through “second-hand” sources) [41]. Cell identities were verified by STR analyses.

Knockdown of PKCδ in MCF7 cells was performed by infection with lentiviral particles. The lentiviral vectors containing short hairpin RNA (shRNA) for PKCδ were obtained from Thermo Scientific (Waltham, MA, USA). A minimum of 5 different shRNA sequences were tested and the two that produced higher knockdown levels were used. Preparation of lentiviral vectors was performed as described previously [42].

To express exogenous P-Rex1-WT and P-Rex1-S^313^A, cells were transfected with pCMV3-myc-P-Rex1-WT and pCMV3-myc-P-Rex1-S^313^A using JetPEI. Transfections were performed according to the instructions provided by the manufacturer. The expression of exogenous P-Rex1 was analyzed by Western blotting with anti-P-Rex antibodies.

### Immunoprecipitation and western blotting

Cultured cells were washed with phosphate-buffered saline (PBS) (NaCl, 137 mM; KCl, 2.7 mM; Na_2_HPO_4_, 8 mM; KH_2_PO_4_, 1.5 mM) and lysed in ice-cold lysis buffer (Tris–HCl [pH 7.0], 20 mM; NaCl, 140 mM; EDTA, 50 mM; glycerol, 10%; Nonidet P-40, 1%; pepstatin, 1 μM; aprotinin, 1 μg/mL; leupeptin, 1 μg/mL; phenylmethyl sulfonyl fluoride, 1 mM; sodium orthovanadate, 1 mM). Lysates were centrifuged at 10,000 xg at 4°C for 10 minutes and supernatants were transferred to new tubes with the corresponding antibody and protein A-Sepharose. Immunoprecipitations were performed at 4°C for at least 2 hours. Immune complexes were recovered by a short centrifugation at 10,000 xg for 15 seconds, followed by three washes with 1 mL ice-cold lysis buffer. Samples were then boiled in electrophoresis sample buffer and placed on SDS-PAGE gels at varying acrylamide concentrations, depending on the molecular weight of the proteins to be analyzed. After electrophoresis, the separated proteins in the gel were transferred to polyvinylidene difluoride membranes (PVDF) (Millipore Corporation, Bedford, MA, USA). Membranes were blocked in TBST (Tris [pH 7.5], 100 mM; NaCl, 150 mM; Tween 20, 0.05%) containing 1% BSA or 5% skimmed milk for 1-3 hours and then incubated with the corresponding antibody for 2-16 hours. After washing three times with TBST during 10 minutes, membranes were incubated with HRP-conjugated anti-mouse or anti-rabbit secondary antibodies for 30 minutes. After the secondary antibody, the membranes were washed three times with TBST and the bands were visualized by using enhanced chemiluminescence [43].

Quantitation of the different bands in western blot was performed by using the NIH Image 1.61 software. The graphics represent the relative protein level. Biochemical assays were repeated at least twice.

### Rac activity

Rac activity was evaluated using a G-LISA Rac activation assay Biochem Kit (Cytoskeleton, Denver, CO, USA) following the manufacturer's instructions. Data are plotted as mean±s.d of duplicates from an experiment which was repeated at least twice.

### Plasmids

The pCMV3 plasmid containing wild type human P-Rex1 tagged with myc was provided by Dr. Heidi C. E. Welch (Babraham Research Campus, Cambridge, UK). The P-Rex1 phosphorylation mutant (S^313^A) was generated as described [4].

### PKCδ in vitro kinase assay

The PKCδ kinase assay was performed as described in the assay procedure in Sigma, Product Code P 6862, with slight modifications. The reaction mix was as follows: HEPES (pH 7.4), 20 mM; MgCl_2_, 10 mM; EGTA, 0.1 mM; ATP, 100 μM; Lipid Mix (phospatidylserine, 12 μg; diacylglycerol, 1.2 μg; in resuspension buffer: HEPES [pH 7.4], 10 mM; Triton X-100, 0.3%); distilled water until 60 μl.

As substrate of the kinase (PKCδ), P-Rex1 immunoprecipitated from MCF7 cells was used. MCF7 cells were lysed and 1 mg of protein was immunoprecipitated with the anti-pan-P-Rex1 antibody. The immunocomplex were washed three times with lysis buffer and twice with water. 60 μl of the reaction mix was added into each immunocomplex and these were placed at 30°C. Then it was added 2 μl of diluted enzyme (PKCδ 50 ng/μl in dilution buffer: HEPES [pH 7.4], 10 mM; DTT, 5 mM; Triton X-100, 0.01%) to each assay tube and the reaction was continued up to 10 minutes. The reaction was stopped by adding loading buffer 4X containing β-mercaptoethanol (20 μl). The reaction of kinase assay was evaluated in Western blot using the anti-phospho-P-Rex1 antibodies.

### Microarray RNA analyses

Gene expression studies were performed as described [33]. Briefly, RNA was extracted and purified with the PureLinkTM Micro-to-Midi kit (Invitrogen, Paisley, UK). cDNA and biotinylated cRNA were synthesized using a T7-polyT primer and the BioArray RNA labeling kit (Enzo, NY, USA), respectively. Labeled RNA was hybridized to Human Gene 1.0 ST oligonucleotide arrays (Affymetrix, CA, USA). For the microarray data analysis, the .CEL files (in triplicate) were imported into the dChip software 19, and expression levels of the different PKC isoforms analysed. Levels of expression (arbitrary units) of the different PKC isoforms were plotted.

### Bioinformatic studies

The sequence in FASTA format of P-Rex1 was introduced in the NetPhos web page (http://www.cbs.dtu.dk/services/NetPhosK/). The NetPhosK program produces neural network predictions of kinase specific eukaryotic protein phosphorylation sites. Currently NetPhosK covers the following kinases: PKA, PKC, PKG, CKII, Cdc2, CaM-II, ATM, DNA PK, Cdk5, p38 MAPK, GSK3, CKI, PKB, RSK, INSR, EGFR and Src [30]. Similarly, the sequence in FASTA format of P-Rex1 was introduced in the Scansite web page. Scansite searches for motifs within proteins that are likely to be phosphorylated by specific protein kinases or bind to domains such as SH2 domains, 14-3-3 domains or PDZ domains (http://scansite3.mit.edu/#home) [31].

### Statistical analyses

Comparisons of continuous variables between two groups were performed using a two-sided Student's *t* test. Differences were considered to be statistically significant when *p*-values were less than 0.05. Statistical data are presented as the mean±s.d. All data were analyzed using the statistical software SPSS 21.0 (SPSS Inc., Chicago, IL).

## SUPPLEMENTARY MATERIALS TABLE




