# Supplementary material for: Multisite phosphorylation of P-Rex1 by protein kinase C
Source: Oncotarget. 2016 Oct 24;7(47):77937–49. doi: 10.18632/oncotarget.12846 (PMC5363633; doi:10.18632/oncotarget.12846)
Supplement: Supplementary file 1 [file oncotarget-07-77937-s001.pdf]

Multisite phosphorylation of P-Rex1 by protein kinase C

SUPPLEMENTARY TABLE

Supplementay Table S1: Scansite results of P-Rex1.

See Supplementary File 1
