# Supplementary material for: Multisite phosphorylation of P-Rex1 by protein kinase C
Source: Oncotarget. 2016 Oct 24;7(47):77937–49. doi: 10.18632/oncotarget.12846 (PMC5363633; doi:10.18632/oncotarget.12846)
Supplement: Supplementary file 2 [file oncotarget-07-77937-s002.docx]

Supplementary Table 1. Scansite results of P-Rex1

| **Tyrosine kinase group (Y_kin)** | | | | |
| --- | --- | --- | --- | --- |
| **Itk Kinase** | | | Gene Card [**ITK**](http://www.genecards.org/cgi-bin/carddisp.pl?gene=ITK) | |
| Site | Score | Percentile | Sequence | SA |
| Y325 | [0.3050](http://scansite.mit.edu/sitestats.phtml?site=Y325&position=325&score=0.3050&percentile=0.026&zscore=-3.442&sequence=KSINGSLYIFRGRIN&protein=REX&motif=Itk_Kin&source=histograms&db=_SWS_&class=VRT) | 0.026 % | [KSINGSL**Y**IFRGRIN](http://scansite.mit.edu/showseq.phtml?prot_id=REX&motif=Itk_Kin&protlength=1659&site=325&protein=XMEAPSGSEPGGDGAGDCAHPDPRAPGAAAPSSGPGPCAAARESERQLRLRLCVLNEILGTERDYVGTLRFLQSAFLHRIRQNVADSVEKGLTEENVKVLFSNIEDILEVHKDFLAALEYCLHPEPQSQHELGNVFLKFKDKFCVYEEYCSNHEKALRLLVELNKIPTVRAFLLSCMLLGGRKTTDIPLEGYLLSPIQRICKYPLLLKELAKRTPGKHPDHPAVQSALQAMKTVCSNINETKRQMEKLEALEQLQSHIEGWEGSNLTDICTQLLLQGTLLKISAGNIQERAFFLFDNLLVYCKRKSRVTGSKKSTKRTKSINGSLYIFRGRINTEVMEVENVEDGTADYHSNGYTVTNGWKIHNTAKNKWFVCMAKTAEEKQKWLDAIIREREQRESLKLGMERDAYVMIAEKGEKLYHMMMNKKVNLIKDRRRKLSTVPKCFLGNEFVAWLLEIGEISKTEEGVNLGQALLENGIIHHVSDKHQFKNEQVMYRFRYDDGTYKARSELEDIMSKGVRLYCRLHSLYTPVIKDRDYHLKTYKSVLPGSKLVDWLLAQGDCQTREEAVALGVGLCNNGFMHHVLEKSEFRDESQYFRFHADEEMEGTSSKNKQLRNDFKLVENILAKRLLILPQEEDYGFDIEEKNKAVVVKSVQRGSLAEVAGLQVGRKIYSINEDLVFLRPFSEVESILNQSFCSRRPLRLLVATKAKEIIKIPDQPDTLCFQIRGAAPPYVYAVGRGSEAMAAGLCAGQCILKVNGSNVMNDGAPEVLEHFQAFRSRREEALGLYQWIYHTHEDAQEARASQEASTEDPSGEQAQEEDQADSAFPLLSLGPRLSLCEDSPMVTLTVDNVHLEHGVVYEYVSTAGVRCHVLEKIVEPRGCFGLTAKILEAFAANDSVFVENCRRLMALSSAIVTMPHFEFRNICDTKLESIGQRIACYQEFAAQLKSRVSPPFKQAPLEPHPLCGLDFCPTNCHINLMEVSYPKTTPSVGRSFSIRFGRKPSLIGLDPEQGHLNPMSYTQHCITTMAAPSWKCLPAAEGDPQGQGLHDGSFGPASGTLGQEDRGLSFLLKQEDREIQDAYLQLFTKLDVALKEMKQYVTQINRLLSTITEPTSGGSCDASLAEEASSLPLVSEESEMDRSDHGGIKKVCFKVAEEDQEDSGHDTMSYRDSYSECNSNRDSVLSYTSVRSNSSYLGSDEMGSGDELPCDMRIPSDKQDKLHGCLEHLFNQVDSINALLKGPVMSRAFEETKHFPMNHSLQEFKQKEECTIRGRSLIQISIQEDPWNLPNSIKTLVDNIQRYVEDGKNQLLLALLKCTDTELQLRRDAIFCQALVAAVCTFSKQLLAALGYRYNNNGEYEESSRDASRKWLEQVAATGVLLHCQSLLSPATVKEERTMLEDIWVTLSELDNVTFSFKQLDENYVANTNVFYHIEGSRQALKVIFYLDSYHFSKLPSRLEGGASLRLHTALFTKVLENVEGLPSPGSQAAEDLQQDINAQSLEKVQQYYRKLRAFYLERSNLPTDASTTAVKIDQLIRPINALDELCRLMKSFVHPKPGAAGSVGAGLIPISSELCYRLGACQMVMCGTGMQRSTLSVSLEQAAILARSHGLLPKCIMQATDIMRKQGPRVEILAKNLRVKDQMPQGAPRLYRLCQPPVDGDL&sequence=KSINGSLYIFRGRIN) | 0.472 |
| **Insulin Receptor Kinase** | | | Gene Card [**INSR**](http://www.genecards.org/cgi-bin/carddisp.pl?gene=INSR) | |
| Site | Score | Percentile | Sequence | SA |
| Y417 | [0.5030](http://scansite.mit.edu/sitestats.phtml?site=Y417&position=417&score=0.5030&percentile=0.088&zscore=-3.566&sequence=AEKGEKLYHMMMNKK&protein=REX&motif=InsR_Kin&source=histograms&db=_SWS_&class=VRT) | 0.088 % | [AEKGEKL**Y**HMMMNKK](http://scansite.mit.edu/showseq.phtml?prot_id=REX&motif=InsR_Kin&protlength=1659&site=417&protein=XMEAPSGSEPGGDGAGDCAHPDPRAPGAAAPSSGPGPCAAARESERQLRLRLCVLNEILGTERDYVGTLRFLQSAFLHRIRQNVADSVEKGLTEENVKVLFSNIEDILEVHKDFLAALEYCLHPEPQSQHELGNVFLKFKDKFCVYEEYCSNHEKALRLLVELNKIPTVRAFLLSCMLLGGRKTTDIPLEGYLLSPIQRICKYPLLLKELAKRTPGKHPDHPAVQSALQAMKTVCSNINETKRQMEKLEALEQLQSHIEGWEGSNLTDICTQLLLQGTLLKISAGNIQERAFFLFDNLLVYCKRKSRVTGSKKSTKRTKSINGSLYIFRGRINTEVMEVENVEDGTADYHSNGYTVTNGWKIHNTAKNKWFVCMAKTAEEKQKWLDAIIREREQRESLKLGMERDAYVMIAEKGEKLYHMMMNKKVNLIKDRRRKLSTVPKCFLGNEFVAWLLEIGEISKTEEGVNLGQALLENGIIHHVSDKHQFKNEQVMYRFRYDDGTYKARSELEDIMSKGVRLYCRLHSLYTPVIKDRDYHLKTYKSVLPGSKLVDWLLAQGDCQTREEAVALGVGLCNNGFMHHVLEKSEFRDESQYFRFHADEEMEGTSSKNKQLRNDFKLVENILAKRLLILPQEEDYGFDIEEKNKAVVVKSVQRGSLAEVAGLQVGRKIYSINEDLVFLRPFSEVESILNQSFCSRRPLRLLVATKAKEIIKIPDQPDTLCFQIRGAAPPYVYAVGRGSEAMAAGLCAGQCILKVNGSNVMNDGAPEVLEHFQAFRSRREEALGLYQWIYHTHEDAQEARASQEASTEDPSGEQAQEEDQADSAFPLLSLGPRLSLCEDSPMVTLTVDNVHLEHGVVYEYVSTAGVRCHVLEKIVEPRGCFGLTAKILEAFAANDSVFVENCRRLMALSSAIVTMPHFEFRNICDTKLESIGQRIACYQEFAAQLKSRVSPPFKQAPLEPHPLCGLDFCPTNCHINLMEVSYPKTTPSVGRSFSIRFGRKPSLIGLDPEQGHLNPMSYTQHCITTMAAPSWKCLPAAEGDPQGQGLHDGSFGPASGTLGQEDRGLSFLLKQEDREIQDAYLQLFTKLDVALKEMKQYVTQINRLLSTITEPTSGGSCDASLAEEASSLPLVSEESEMDRSDHGGIKKVCFKVAEEDQEDSGHDTMSYRDSYSECNSNRDSVLSYTSVRSNSSYLGSDEMGSGDELPCDMRIPSDKQDKLHGCLEHLFNQVDSINALLKGPVMSRAFEETKHFPMNHSLQEFKQKEECTIRGRSLIQISIQEDPWNLPNSIKTLVDNIQRYVEDGKNQLLLALLKCTDTELQLRRDAIFCQALVAAVCTFSKQLLAALGYRYNNNGEYEESSRDASRKWLEQVAATGVLLHCQSLLSPATVKEERTMLEDIWVTLSELDNVTFSFKQLDENYVANTNVFYHIEGSRQALKVIFYLDSYHFSKLPSRLEGGASLRLHTALFTKVLENVEGLPSPGSQAAEDLQQDINAQSLEKVQQYYRKLRAFYLERSNLPTDASTTAVKIDQLIRPINALDELCRLMKSFVHPKPGAAGSVGAGLIPISSELCYRLGACQMVMCGTGMQRSTLSVSLEQAAILARSHGLLPKCIMQATDIMRKQGPRVEILAKNLRVKDQMPQGAPRLYRLCQPPVDGDL&sequence=AEKGEKLYHMMMNKK) | 0.789 |
| **Src homology 2 group (SH2)** | | | | |
| **Src SH2** | | | Gene Card [**SRC**](http://www.genecards.org/cgi-bin/carddisp.pl?gene=SRC) | |
| Site | Score | Percentile | Sequence | SA |
| Y857 | [0.2376](http://scansite.mit.edu/sitestats.phtml?site=Y857&position=857&score=0.2376&percentile=0.012&zscore=-4.431&sequence=HLEHGVVYEYVSTAG&protein=REX&motif=Src_SH2&source=histograms&db=_SWS_&class=VRT) | 0.012 % | [HLEHGVV**Y**EYVSTAG](http://scansite.mit.edu/showseq.phtml?prot_id=REX&motif=Src_SH2&protlength=1659&site=857&protein=XMEAPSGSEPGGDGAGDCAHPDPRAPGAAAPSSGPGPCAAARESERQLRLRLCVLNEILGTERDYVGTLRFLQSAFLHRIRQNVADSVEKGLTEENVKVLFSNIEDILEVHKDFLAALEYCLHPEPQSQHELGNVFLKFKDKFCVYEEYCSNHEKALRLLVELNKIPTVRAFLLSCMLLGGRKTTDIPLEGYLLSPIQRICKYPLLLKELAKRTPGKHPDHPAVQSALQAMKTVCSNINETKRQMEKLEALEQLQSHIEGWEGSNLTDICTQLLLQGTLLKISAGNIQERAFFLFDNLLVYCKRKSRVTGSKKSTKRTKSINGSLYIFRGRINTEVMEVENVEDGTADYHSNGYTVTNGWKIHNTAKNKWFVCMAKTAEEKQKWLDAIIREREQRESLKLGMERDAYVMIAEKGEKLYHMMMNKKVNLIKDRRRKLSTVPKCFLGNEFVAWLLEIGEISKTEEGVNLGQALLENGIIHHVSDKHQFKNEQVMYRFRYDDGTYKARSELEDIMSKGVRLYCRLHSLYTPVIKDRDYHLKTYKSVLPGSKLVDWLLAQGDCQTREEAVALGVGLCNNGFMHHVLEKSEFRDESQYFRFHADEEMEGTSSKNKQLRNDFKLVENILAKRLLILPQEEDYGFDIEEKNKAVVVKSVQRGSLAEVAGLQVGRKIYSINEDLVFLRPFSEVESILNQSFCSRRPLRLLVATKAKEIIKIPDQPDTLCFQIRGAAPPYVYAVGRGSEAMAAGLCAGQCILKVNGSNVMNDGAPEVLEHFQAFRSRREEALGLYQWIYHTHEDAQEARASQEASTEDPSGEQAQEEDQADSAFPLLSLGPRLSLCEDSPMVTLTVDNVHLEHGVVYEYVSTAGVRCHVLEKIVEPRGCFGLTAKILEAFAANDSVFVENCRRLMALSSAIVTMPHFEFRNICDTKLESIGQRIACYQEFAAQLKSRVSPPFKQAPLEPHPLCGLDFCPTNCHINLMEVSYPKTTPSVGRSFSIRFGRKPSLIGLDPEQGHLNPMSYTQHCITTMAAPSWKCLPAAEGDPQGQGLHDGSFGPASGTLGQEDRGLSFLLKQEDREIQDAYLQLFTKLDVALKEMKQYVTQINRLLSTITEPTSGGSCDASLAEEASSLPLVSEESEMDRSDHGGIKKVCFKVAEEDQEDSGHDTMSYRDSYSECNSNRDSVLSYTSVRSNSSYLGSDEMGSGDELPCDMRIPSDKQDKLHGCLEHLFNQVDSINALLKGPVMSRAFEETKHFPMNHSLQEFKQKEECTIRGRSLIQISIQEDPWNLPNSIKTLVDNIQRYVEDGKNQLLLALLKCTDTELQLRRDAIFCQALVAAVCTFSKQLLAALGYRYNNNGEYEESSRDASRKWLEQVAATGVLLHCQSLLSPATVKEERTMLEDIWVTLSELDNVTFSFKQLDENYVANTNVFYHIEGSRQALKVIFYLDSYHFSKLPSRLEGGASLRLHTALFTKVLENVEGLPSPGSQAAEDLQQDINAQSLEKVQQYYRKLRAFYLERSNLPTDASTTAVKIDQLIRPINALDELCRLMKSFVHPKPGAAGSVGAGLIPISSELCYRLGACQMVMCGTGMQRSTLSVSLEQAAILARSHGLLPKCIMQATDIMRKQGPRVEILAKNLRVKDQMPQGAPRLYRLCQPPVDGDL&sequence=HLEHGVVYEYVSTAG) | 0.398 |
| **Lck SH2** | | | Gene Card [**LCK**](http://www.genecards.org/cgi-bin/carddisp.pl?gene=LCK) | |
| Site | Score | Percentile | Sequence | SA |
| Y857 | [0.2917](http://scansite.mit.edu/sitestats.phtml?site=Y857&position=857&score=0.2917&percentile=0.041&zscore=-4.015&sequence=HLEHGVVYEYVSTAG&protein=REX&motif=Lck_SH2&source=histograms&db=_SWS_&class=VRT) | 0.041 % | [HLEHGVV**Y**EYVSTAG](http://scansite.mit.edu/showseq.phtml?prot_id=REX&motif=Lck_SH2&protlength=1659&site=857&protein=XMEAPSGSEPGGDGAGDCAHPDPRAPGAAAPSSGPGPCAAARESERQLRLRLCVLNEILGTERDYVGTLRFLQSAFLHRIRQNVADSVEKGLTEENVKVLFSNIEDILEVHKDFLAALEYCLHPEPQSQHELGNVFLKFKDKFCVYEEYCSNHEKALRLLVELNKIPTVRAFLLSCMLLGGRKTTDIPLEGYLLSPIQRICKYPLLLKELAKRTPGKHPDHPAVQSALQAMKTVCSNINETKRQMEKLEALEQLQSHIEGWEGSNLTDICTQLLLQGTLLKISAGNIQERAFFLFDNLLVYCKRKSRVTGSKKSTKRTKSINGSLYIFRGRINTEVMEVENVEDGTADYHSNGYTVTNGWKIHNTAKNKWFVCMAKTAEEKQKWLDAIIREREQRESLKLGMERDAYVMIAEKGEKLYHMMMNKKVNLIKDRRRKLSTVPKCFLGNEFVAWLLEIGEISKTEEGVNLGQALLENGIIHHVSDKHQFKNEQVMYRFRYDDGTYKARSELEDIMSKGVRLYCRLHSLYTPVIKDRDYHLKTYKSVLPGSKLVDWLLAQGDCQTREEAVALGVGLCNNGFMHHVLEKSEFRDESQYFRFHADEEMEGTSSKNKQLRNDFKLVENILAKRLLILPQEEDYGFDIEEKNKAVVVKSVQRGSLAEVAGLQVGRKIYSINEDLVFLRPFSEVESILNQSFCSRRPLRLLVATKAKEIIKIPDQPDTLCFQIRGAAPPYVYAVGRGSEAMAAGLCAGQCILKVNGSNVMNDGAPEVLEHFQAFRSRREEALGLYQWIYHTHEDAQEARASQEASTEDPSGEQAQEEDQADSAFPLLSLGPRLSLCEDSPMVTLTVDNVHLEHGVVYEYVSTAGVRCHVLEKIVEPRGCFGLTAKILEAFAANDSVFVENCRRLMALSSAIVTMPHFEFRNICDTKLESIGQRIACYQEFAAQLKSRVSPPFKQAPLEPHPLCGLDFCPTNCHINLMEVSYPKTTPSVGRSFSIRFGRKPSLIGLDPEQGHLNPMSYTQHCITTMAAPSWKCLPAAEGDPQGQGLHDGSFGPASGTLGQEDRGLSFLLKQEDREIQDAYLQLFTKLDVALKEMKQYVTQINRLLSTITEPTSGGSCDASLAEEASSLPLVSEESEMDRSDHGGIKKVCFKVAEEDQEDSGHDTMSYRDSYSECNSNRDSVLSYTSVRSNSSYLGSDEMGSGDELPCDMRIPSDKQDKLHGCLEHLFNQVDSINALLKGPVMSRAFEETKHFPMNHSLQEFKQKEECTIRGRSLIQISIQEDPWNLPNSIKTLVDNIQRYVEDGKNQLLLALLKCTDTELQLRRDAIFCQALVAAVCTFSKQLLAALGYRYNNNGEYEESSRDASRKWLEQVAATGVLLHCQSLLSPATVKEERTMLEDIWVTLSELDNVTFSFKQLDENYVANTNVFYHIEGSRQALKVIFYLDSYHFSKLPSRLEGGASLRLHTALFTKVLENVEGLPSPGSQAAEDLQQDINAQSLEKVQQYYRKLRAFYLERSNLPTDASTTAVKIDQLIRPINALDELCRLMKSFVHPKPGAAGSVGAGLIPISSELCYRLGACQMVMCGTGMQRSTLSVSLEQAAILARSHGLLPKCIMQATDIMRKQGPRVEILAKNLRVKDQMPQGAPRLYRLCQPPVDGDL&sequence=HLEHGVVYEYVSTAG) | 0.398 |
| **Basophilic serine/threonine kinase group (Baso_ST_kin)** | | | | |
| **PKC epsilon** | | | Gene Card [**PRKCE**](http://www.genecards.org/cgi-bin/carddisp.pl?gene=PRKCE) | |
| Site | Score | Percentile | Sequence | SA |
| S655 | [0.3201](http://scansite.mit.edu/sitestats.phtml?site=S655&position=655&score=0.3201&percentile=0.019&zscore=-3.644&sequence=VKSVQRGSLAEVAGL&protein=REX&motif=PKC_epsilon&source=histograms&db=_SWS_&class=VRT) | 0.019 % | [VKSVQRG**S**LAEVAGL](http://scansite.mit.edu/showseq.phtml?prot_id=REX&motif=PKC_epsilon&protlength=1659&site=655&protein=XMEAPSGSEPGGDGAGDCAHPDPRAPGAAAPSSGPGPCAAARESERQLRLRLCVLNEILGTERDYVGTLRFLQSAFLHRIRQNVADSVEKGLTEENVKVLFSNIEDILEVHKDFLAALEYCLHPEPQSQHELGNVFLKFKDKFCVYEEYCSNHEKALRLLVELNKIPTVRAFLLSCMLLGGRKTTDIPLEGYLLSPIQRICKYPLLLKELAKRTPGKHPDHPAVQSALQAMKTVCSNINETKRQMEKLEALEQLQSHIEGWEGSNLTDICTQLLLQGTLLKISAGNIQERAFFLFDNLLVYCKRKSRVTGSKKSTKRTKSINGSLYIFRGRINTEVMEVENVEDGTADYHSNGYTVTNGWKIHNTAKNKWFVCMAKTAEEKQKWLDAIIREREQRESLKLGMERDAYVMIAEKGEKLYHMMMNKKVNLIKDRRRKLSTVPKCFLGNEFVAWLLEIGEISKTEEGVNLGQALLENGIIHHVSDKHQFKNEQVMYRFRYDDGTYKARSELEDIMSKGVRLYCRLHSLYTPVIKDRDYHLKTYKSVLPGSKLVDWLLAQGDCQTREEAVALGVGLCNNGFMHHVLEKSEFRDESQYFRFHADEEMEGTSSKNKQLRNDFKLVENILAKRLLILPQEEDYGFDIEEKNKAVVVKSVQRGSLAEVAGLQVGRKIYSINEDLVFLRPFSEVESILNQSFCSRRPLRLLVATKAKEIIKIPDQPDTLCFQIRGAAPPYVYAVGRGSEAMAAGLCAGQCILKVNGSNVMNDGAPEVLEHFQAFRSRREEALGLYQWIYHTHEDAQEARASQEASTEDPSGEQAQEEDQADSAFPLLSLGPRLSLCEDSPMVTLTVDNVHLEHGVVYEYVSTAGVRCHVLEKIVEPRGCFGLTAKILEAFAANDSVFVENCRRLMALSSAIVTMPHFEFRNICDTKLESIGQRIACYQEFAAQLKSRVSPPFKQAPLEPHPLCGLDFCPTNCHINLMEVSYPKTTPSVGRSFSIRFGRKPSLIGLDPEQGHLNPMSYTQHCITTMAAPSWKCLPAAEGDPQGQGLHDGSFGPASGTLGQEDRGLSFLLKQEDREIQDAYLQLFTKLDVALKEMKQYVTQINRLLSTITEPTSGGSCDASLAEEASSLPLVSEESEMDRSDHGGIKKVCFKVAEEDQEDSGHDTMSYRDSYSECNSNRDSVLSYTSVRSNSSYLGSDEMGSGDELPCDMRIPSDKQDKLHGCLEHLFNQVDSINALLKGPVMSRAFEETKHFPMNHSLQEFKQKEECTIRGRSLIQISIQEDPWNLPNSIKTLVDNIQRYVEDGKNQLLLALLKCTDTELQLRRDAIFCQALVAAVCTFSKQLLAALGYRYNNNGEYEESSRDASRKWLEQVAATGVLLHCQSLLSPATVKEERTMLEDIWVTLSELDNVTFSFKQLDENYVANTNVFYHIEGSRQALKVIFYLDSYHFSKLPSRLEGGASLRLHTALFTKVLENVEGLPSPGSQAAEDLQQDINAQSLEKVQQYYRKLRAFYLERSNLPTDASTTAVKIDQLIRPINALDELCRLMKSFVHPKPGAAGSVGAGLIPISSELCYRLGACQMVMCGTGMQRSTLSVSLEQAAILARSHGLLPKCIMQATDIMRKQGPRVEILAKNLRVKDQMPQGAPRLYRLCQPPVDGDL&sequence=VKSVQRGSLAEVAGL) | 0.859 |
| **PKC alpha/beta/gamma** | | | Gene Card [**PRKCA**](http://www.genecards.org/cgi-bin/carddisp.pl?gene=PRKCA) | |
| Site | Score | Percentile | Sequence | SA |
| S313 | [0.3353](http://scansite.mit.edu/sitestats.phtml?site=S313&position=313&score=0.3353&percentile=0.038&zscore=-3.970&sequence=RVTGSKKSTKRTKSI&protein=REX&motif=PKC_common&source=histograms&db=_SWS_&class=VRT) | 0.038 % | [RVTGSKK**S**TKRTKSI](http://scansite.mit.edu/showseq.phtml?prot_id=REX&motif=PKC_common&protlength=1659&site=313&protein=XMEAPSGSEPGGDGAGDCAHPDPRAPGAAAPSSGPGPCAAARESERQLRLRLCVLNEILGTERDYVGTLRFLQSAFLHRIRQNVADSVEKGLTEENVKVLFSNIEDILEVHKDFLAALEYCLHPEPQSQHELGNVFLKFKDKFCVYEEYCSNHEKALRLLVELNKIPTVRAFLLSCMLLGGRKTTDIPLEGYLLSPIQRICKYPLLLKELAKRTPGKHPDHPAVQSALQAMKTVCSNINETKRQMEKLEALEQLQSHIEGWEGSNLTDICTQLLLQGTLLKISAGNIQERAFFLFDNLLVYCKRKSRVTGSKKSTKRTKSINGSLYIFRGRINTEVMEVENVEDGTADYHSNGYTVTNGWKIHNTAKNKWFVCMAKTAEEKQKWLDAIIREREQRESLKLGMERDAYVMIAEKGEKLYHMMMNKKVNLIKDRRRKLSTVPKCFLGNEFVAWLLEIGEISKTEEGVNLGQALLENGIIHHVSDKHQFKNEQVMYRFRYDDGTYKARSELEDIMSKGVRLYCRLHSLYTPVIKDRDYHLKTYKSVLPGSKLVDWLLAQGDCQTREEAVALGVGLCNNGFMHHVLEKSEFRDESQYFRFHADEEMEGTSSKNKQLRNDFKLVENILAKRLLILPQEEDYGFDIEEKNKAVVVKSVQRGSLAEVAGLQVGRKIYSINEDLVFLRPFSEVESILNQSFCSRRPLRLLVATKAKEIIKIPDQPDTLCFQIRGAAPPYVYAVGRGSEAMAAGLCAGQCILKVNGSNVMNDGAPEVLEHFQAFRSRREEALGLYQWIYHTHEDAQEARASQEASTEDPSGEQAQEEDQADSAFPLLSLGPRLSLCEDSPMVTLTVDNVHLEHGVVYEYVSTAGVRCHVLEKIVEPRGCFGLTAKILEAFAANDSVFVENCRRLMALSSAIVTMPHFEFRNICDTKLESIGQRIACYQEFAAQLKSRVSPPFKQAPLEPHPLCGLDFCPTNCHINLMEVSYPKTTPSVGRSFSIRFGRKPSLIGLDPEQGHLNPMSYTQHCITTMAAPSWKCLPAAEGDPQGQGLHDGSFGPASGTLGQEDRGLSFLLKQEDREIQDAYLQLFTKLDVALKEMKQYVTQINRLLSTITEPTSGGSCDASLAEEASSLPLVSEESEMDRSDHGGIKKVCFKVAEEDQEDSGHDTMSYRDSYSECNSNRDSVLSYTSVRSNSSYLGSDEMGSGDELPCDMRIPSDKQDKLHGCLEHLFNQVDSINALLKGPVMSRAFEETKHFPMNHSLQEFKQKEECTIRGRSLIQISIQEDPWNLPNSIKTLVDNIQRYVEDGKNQLLLALLKCTDTELQLRRDAIFCQALVAAVCTFSKQLLAALGYRYNNNGEYEESSRDASRKWLEQVAATGVLLHCQSLLSPATVKEERTMLEDIWVTLSELDNVTFSFKQLDENYVANTNVFYHIEGSRQALKVIFYLDSYHFSKLPSRLEGGASLRLHTALFTKVLENVEGLPSPGSQAAEDLQQDINAQSLEKVQQYYRKLRAFYLERSNLPTDASTTAVKIDQLIRPINALDELCRLMKSFVHPKPGAAGSVGAGLIPISSELCYRLGACQMVMCGTGMQRSTLSVSLEQAAILARSHGLLPKCIMQATDIMRKQGPRVEILAKNLRVKDQMPQGAPRLYRLCQPPVDGDL&sequence=RVTGSKKSTKRTKSI) | 6.943 |
| **PKC alpha/beta/gamma** | | | Gene Card [**PRKCA**](http://www.genecards.org/cgi-bin/carddisp.pl?gene=PRKCA) | |
| Site | Score | Percentile | Sequence | SA |
| S310 | [0.3837](http://scansite.mit.edu/sitestats.phtml?site=S310&position=310&score=0.3837&percentile=0.130&zscore=-3.496&sequence=RKSRVTGSKKSTKRT&protein=REX&motif=PKC_common&source=histograms&db=_SWS_&class=VRT) | 0.130 % | [RKSRVTG**S**KKSTKRT](http://scansite.mit.edu/showseq.phtml?prot_id=REX&motif=PKC_common&protlength=1659&site=310&protein=XMEAPSGSEPGGDGAGDCAHPDPRAPGAAAPSSGPGPCAAARESERQLRLRLCVLNEILGTERDYVGTLRFLQSAFLHRIRQNVADSVEKGLTEENVKVLFSNIEDILEVHKDFLAALEYCLHPEPQSQHELGNVFLKFKDKFCVYEEYCSNHEKALRLLVELNKIPTVRAFLLSCMLLGGRKTTDIPLEGYLLSPIQRICKYPLLLKELAKRTPGKHPDHPAVQSALQAMKTVCSNINETKRQMEKLEALEQLQSHIEGWEGSNLTDICTQLLLQGTLLKISAGNIQERAFFLFDNLLVYCKRKSRVTGSKKSTKRTKSINGSLYIFRGRINTEVMEVENVEDGTADYHSNGYTVTNGWKIHNTAKNKWFVCMAKTAEEKQKWLDAIIREREQRESLKLGMERDAYVMIAEKGEKLYHMMMNKKVNLIKDRRRKLSTVPKCFLGNEFVAWLLEIGEISKTEEGVNLGQALLENGIIHHVSDKHQFKNEQVMYRFRYDDGTYKARSELEDIMSKGVRLYCRLHSLYTPVIKDRDYHLKTYKSVLPGSKLVDWLLAQGDCQTREEAVALGVGLCNNGFMHHVLEKSEFRDESQYFRFHADEEMEGTSSKNKQLRNDFKLVENILAKRLLILPQEEDYGFDIEEKNKAVVVKSVQRGSLAEVAGLQVGRKIYSINEDLVFLRPFSEVESILNQSFCSRRPLRLLVATKAKEIIKIPDQPDTLCFQIRGAAPPYVYAVGRGSEAMAAGLCAGQCILKVNGSNVMNDGAPEVLEHFQAFRSRREEALGLYQWIYHTHEDAQEARASQEASTEDPSGEQAQEEDQADSAFPLLSLGPRLSLCEDSPMVTLTVDNVHLEHGVVYEYVSTAGVRCHVLEKIVEPRGCFGLTAKILEAFAANDSVFVENCRRLMALSSAIVTMPHFEFRNICDTKLESIGQRIACYQEFAAQLKSRVSPPFKQAPLEPHPLCGLDFCPTNCHINLMEVSYPKTTPSVGRSFSIRFGRKPSLIGLDPEQGHLNPMSYTQHCITTMAAPSWKCLPAAEGDPQGQGLHDGSFGPASGTLGQEDRGLSFLLKQEDREIQDAYLQLFTKLDVALKEMKQYVTQINRLLSTITEPTSGGSCDASLAEEASSLPLVSEESEMDRSDHGGIKKVCFKVAEEDQEDSGHDTMSYRDSYSECNSNRDSVLSYTSVRSNSSYLGSDEMGSGDELPCDMRIPSDKQDKLHGCLEHLFNQVDSINALLKGPVMSRAFEETKHFPMNHSLQEFKQKEECTIRGRSLIQISIQEDPWNLPNSIKTLVDNIQRYVEDGKNQLLLALLKCTDTELQLRRDAIFCQALVAAVCTFSKQLLAALGYRYNNNGEYEESSRDASRKWLEQVAATGVLLHCQSLLSPATVKEERTMLEDIWVTLSELDNVTFSFKQLDENYVANTNVFYHIEGSRQALKVIFYLDSYHFSKLPSRLEGGASLRLHTALFTKVLENVEGLPSPGSQAAEDLQQDINAQSLEKVQQYYRKLRAFYLERSNLPTDASTTAVKIDQLIRPINALDELCRLMKSFVHPKPGAAGSVGAGLIPISSELCYRLGACQMVMCGTGMQRSTLSVSLEQAAILARSHGLLPKCIMQATDIMRKQGPRVEILAKNLRVKDQMPQGAPRLYRLCQPPVDGDL&sequence=RKSRVTGSKKSTKRT) | 2.351 |
| **Calmodulin dependent Kinase 2** | | | Gene Card [**CAMK2G**](http://www.genecards.org/cgi-bin/carddisp.pl?gene=CAMK2G) | |
| Site | Score | Percentile | Sequence | SA |
| S1065 | [0.4078](http://scansite.mit.edu/sitestats.phtml?site=S1065&position=1065&score=0.4078&percentile=0.086&zscore=-3.795&sequence=GQEDRGLSFLLKQED&protein=REX&motif=Cam_Kin2&source=histograms&db=_SWS_&class=VRT) | 0.086 % | [GQEDRGL**S**FLLKQED](http://scansite.mit.edu/showseq.phtml?prot_id=REX&motif=Cam_Kin2&protlength=1659&site=1065&protein=XMEAPSGSEPGGDGAGDCAHPDPRAPGAAAPSSGPGPCAAARESERQLRLRLCVLNEILGTERDYVGTLRFLQSAFLHRIRQNVADSVEKGLTEENVKVLFSNIEDILEVHKDFLAALEYCLHPEPQSQHELGNVFLKFKDKFCVYEEYCSNHEKALRLLVELNKIPTVRAFLLSCMLLGGRKTTDIPLEGYLLSPIQRICKYPLLLKELAKRTPGKHPDHPAVQSALQAMKTVCSNINETKRQMEKLEALEQLQSHIEGWEGSNLTDICTQLLLQGTLLKISAGNIQERAFFLFDNLLVYCKRKSRVTGSKKSTKRTKSINGSLYIFRGRINTEVMEVENVEDGTADYHSNGYTVTNGWKIHNTAKNKWFVCMAKTAEEKQKWLDAIIREREQRESLKLGMERDAYVMIAEKGEKLYHMMMNKKVNLIKDRRRKLSTVPKCFLGNEFVAWLLEIGEISKTEEGVNLGQALLENGIIHHVSDKHQFKNEQVMYRFRYDDGTYKARSELEDIMSKGVRLYCRLHSLYTPVIKDRDYHLKTYKSVLPGSKLVDWLLAQGDCQTREEAVALGVGLCNNGFMHHVLEKSEFRDESQYFRFHADEEMEGTSSKNKQLRNDFKLVENILAKRLLILPQEEDYGFDIEEKNKAVVVKSVQRGSLAEVAGLQVGRKIYSINEDLVFLRPFSEVESILNQSFCSRRPLRLLVATKAKEIIKIPDQPDTLCFQIRGAAPPYVYAVGRGSEAMAAGLCAGQCILKVNGSNVMNDGAPEVLEHFQAFRSRREEALGLYQWIYHTHEDAQEARASQEASTEDPSGEQAQEEDQADSAFPLLSLGPRLSLCEDSPMVTLTVDNVHLEHGVVYEYVSTAGVRCHVLEKIVEPRGCFGLTAKILEAFAANDSVFVENCRRLMALSSAIVTMPHFEFRNICDTKLESIGQRIACYQEFAAQLKSRVSPPFKQAPLEPHPLCGLDFCPTNCHINLMEVSYPKTTPSVGRSFSIRFGRKPSLIGLDPEQGHLNPMSYTQHCITTMAAPSWKCLPAAEGDPQGQGLHDGSFGPASGTLGQEDRGLSFLLKQEDREIQDAYLQLFTKLDVALKEMKQYVTQINRLLSTITEPTSGGSCDASLAEEASSLPLVSEESEMDRSDHGGIKKVCFKVAEEDQEDSGHDTMSYRDSYSECNSNRDSVLSYTSVRSNSSYLGSDEMGSGDELPCDMRIPSDKQDKLHGCLEHLFNQVDSINALLKGPVMSRAFEETKHFPMNHSLQEFKQKEECTIRGRSLIQISIQEDPWNLPNSIKTLVDNIQRYVEDGKNQLLLALLKCTDTELQLRRDAIFCQALVAAVCTFSKQLLAALGYRYNNNGEYEESSRDASRKWLEQVAATGVLLHCQSLLSPATVKEERTMLEDIWVTLSELDNVTFSFKQLDENYVANTNVFYHIEGSRQALKVIFYLDSYHFSKLPSRLEGGASLRLHTALFTKVLENVEGLPSPGSQAAEDLQQDINAQSLEKVQQYYRKLRAFYLERSNLPTDASTTAVKIDQLIRPINALDELCRLMKSFVHPKPGAAGSVGAGLIPISSELCYRLGACQMVMCGTGMQRSTLSVSLEQAAILARSHGLLPKCIMQATDIMRKQGPRVEILAKNLRVKDQMPQGAPRLYRLCQPPVDGDL&sequence=GQEDRGLSFLLKQED) | 0.148 |
| **Clk2 Kinase** | | | Gene Card [**CLK2**](http://www.genecards.org/cgi-bin/carddisp.pl?gene=CLK2) | |
| Site | Score | Percentile | Sequence | SA |
| S1364 | [0.5186](http://scansite.mit.edu/sitestats.phtml?site=S1364&position=1364&score=0.5186&percentile=0.128&zscore=-4.063&sequence=EESSRDASRKWLEQV&protein=REX&motif=Clk2_Kin&source=histograms&db=_SWS_&class=VRT) | 0.128 % | [EESSRDA**S**RKWLEQV](http://scansite.mit.edu/showseq.phtml?prot_id=REX&motif=Clk2_Kin&protlength=1659&site=1364&protein=XMEAPSGSEPGGDGAGDCAHPDPRAPGAAAPSSGPGPCAAARESERQLRLRLCVLNEILGTERDYVGTLRFLQSAFLHRIRQNVADSVEKGLTEENVKVLFSNIEDILEVHKDFLAALEYCLHPEPQSQHELGNVFLKFKDKFCVYEEYCSNHEKALRLLVELNKIPTVRAFLLSCMLLGGRKTTDIPLEGYLLSPIQRICKYPLLLKELAKRTPGKHPDHPAVQSALQAMKTVCSNINETKRQMEKLEALEQLQSHIEGWEGSNLTDICTQLLLQGTLLKISAGNIQERAFFLFDNLLVYCKRKSRVTGSKKSTKRTKSINGSLYIFRGRINTEVMEVENVEDGTADYHSNGYTVTNGWKIHNTAKNKWFVCMAKTAEEKQKWLDAIIREREQRESLKLGMERDAYVMIAEKGEKLYHMMMNKKVNLIKDRRRKLSTVPKCFLGNEFVAWLLEIGEISKTEEGVNLGQALLENGIIHHVSDKHQFKNEQVMYRFRYDDGTYKARSELEDIMSKGVRLYCRLHSLYTPVIKDRDYHLKTYKSVLPGSKLVDWLLAQGDCQTREEAVALGVGLCNNGFMHHVLEKSEFRDESQYFRFHADEEMEGTSSKNKQLRNDFKLVENILAKRLLILPQEEDYGFDIEEKNKAVVVKSVQRGSLAEVAGLQVGRKIYSINEDLVFLRPFSEVESILNQSFCSRRPLRLLVATKAKEIIKIPDQPDTLCFQIRGAAPPYVYAVGRGSEAMAAGLCAGQCILKVNGSNVMNDGAPEVLEHFQAFRSRREEALGLYQWIYHTHEDAQEARASQEASTEDPSGEQAQEEDQADSAFPLLSLGPRLSLCEDSPMVTLTVDNVHLEHGVVYEYVSTAGVRCHVLEKIVEPRGCFGLTAKILEAFAANDSVFVENCRRLMALSSAIVTMPHFEFRNICDTKLESIGQRIACYQEFAAQLKSRVSPPFKQAPLEPHPLCGLDFCPTNCHINLMEVSYPKTTPSVGRSFSIRFGRKPSLIGLDPEQGHLNPMSYTQHCITTMAAPSWKCLPAAEGDPQGQGLHDGSFGPASGTLGQEDRGLSFLLKQEDREIQDAYLQLFTKLDVALKEMKQYVTQINRLLSTITEPTSGGSCDASLAEEASSLPLVSEESEMDRSDHGGIKKVCFKVAEEDQEDSGHDTMSYRDSYSECNSNRDSVLSYTSVRSNSSYLGSDEMGSGDELPCDMRIPSDKQDKLHGCLEHLFNQVDSINALLKGPVMSRAFEETKHFPMNHSLQEFKQKEECTIRGRSLIQISIQEDPWNLPNSIKTLVDNIQRYVEDGKNQLLLALLKCTDTELQLRRDAIFCQALVAAVCTFSKQLLAALGYRYNNNGEYEESSRDASRKWLEQVAATGVLLHCQSLLSPATVKEERTMLEDIWVTLSELDNVTFSFKQLDENYVANTNVFYHIEGSRQALKVIFYLDSYHFSKLPSRLEGGASLRLHTALFTKVLENVEGLPSPGSQAAEDLQQDINAQSLEKVQQYYRKLRAFYLERSNLPTDASTTAVKIDQLIRPINALDELCRLMKSFVHPKPGAAGSVGAGLIPISSELCYRLGACQMVMCGTGMQRSTLSVSLEQAAILARSHGLLPKCIMQATDIMRKQGPRVEILAKNLRVKDQMPQGAPRLYRLCQPPVDGDL&sequence=EESSRDASRKWLEQV) | 2.134 |
| **DNA damage kinase group (DNA_dam_kin)** | | | | |
| **DNA PK** | | | Gene Card [**PRKDC**](http://www.genecards.org/cgi-bin/carddisp.pl?gene=PRKDC) | |
| Site | Score | Percentile | Sequence | SA |
| S801 | [0.3848](http://scansite.mit.edu/sitestats.phtml?site=S801&position=801&score=0.3848&percentile=0.066&zscore=-3.879&sequence=DAQEARASQEASTED&protein=REX&motif=DNA_PK&source=histograms&db=_SWS_&class=VRT) | 0.066 % | [DAQEARA**S**QEASTED](http://scansite.mit.edu/showseq.phtml?prot_id=REX&motif=DNA_PK&protlength=1659&site=801&protein=XMEAPSGSEPGGDGAGDCAHPDPRAPGAAAPSSGPGPCAAARESERQLRLRLCVLNEILGTERDYVGTLRFLQSAFLHRIRQNVADSVEKGLTEENVKVLFSNIEDILEVHKDFLAALEYCLHPEPQSQHELGNVFLKFKDKFCVYEEYCSNHEKALRLLVELNKIPTVRAFLLSCMLLGGRKTTDIPLEGYLLSPIQRICKYPLLLKELAKRTPGKHPDHPAVQSALQAMKTVCSNINETKRQMEKLEALEQLQSHIEGWEGSNLTDICTQLLLQGTLLKISAGNIQERAFFLFDNLLVYCKRKSRVTGSKKSTKRTKSINGSLYIFRGRINTEVMEVENVEDGTADYHSNGYTVTNGWKIHNTAKNKWFVCMAKTAEEKQKWLDAIIREREQRESLKLGMERDAYVMIAEKGEKLYHMMMNKKVNLIKDRRRKLSTVPKCFLGNEFVAWLLEIGEISKTEEGVNLGQALLENGIIHHVSDKHQFKNEQVMYRFRYDDGTYKARSELEDIMSKGVRLYCRLHSLYTPVIKDRDYHLKTYKSVLPGSKLVDWLLAQGDCQTREEAVALGVGLCNNGFMHHVLEKSEFRDESQYFRFHADEEMEGTSSKNKQLRNDFKLVENILAKRLLILPQEEDYGFDIEEKNKAVVVKSVQRGSLAEVAGLQVGRKIYSINEDLVFLRPFSEVESILNQSFCSRRPLRLLVATKAKEIIKIPDQPDTLCFQIRGAAPPYVYAVGRGSEAMAAGLCAGQCILKVNGSNVMNDGAPEVLEHFQAFRSRREEALGLYQWIYHTHEDAQEARASQEASTEDPSGEQAQEEDQADSAFPLLSLGPRLSLCEDSPMVTLTVDNVHLEHGVVYEYVSTAGVRCHVLEKIVEPRGCFGLTAKILEAFAANDSVFVENCRRLMALSSAIVTMPHFEFRNICDTKLESIGQRIACYQEFAAQLKSRVSPPFKQAPLEPHPLCGLDFCPTNCHINLMEVSYPKTTPSVGRSFSIRFGRKPSLIGLDPEQGHLNPMSYTQHCITTMAAPSWKCLPAAEGDPQGQGLHDGSFGPASGTLGQEDRGLSFLLKQEDREIQDAYLQLFTKLDVALKEMKQYVTQINRLLSTITEPTSGGSCDASLAEEASSLPLVSEESEMDRSDHGGIKKVCFKVAEEDQEDSGHDTMSYRDSYSECNSNRDSVLSYTSVRSNSSYLGSDEMGSGDELPCDMRIPSDKQDKLHGCLEHLFNQVDSINALLKGPVMSRAFEETKHFPMNHSLQEFKQKEECTIRGRSLIQISIQEDPWNLPNSIKTLVDNIQRYVEDGKNQLLLALLKCTDTELQLRRDAIFCQALVAAVCTFSKQLLAALGYRYNNNGEYEESSRDASRKWLEQVAATGVLLHCQSLLSPATVKEERTMLEDIWVTLSELDNVTFSFKQLDENYVANTNVFYHIEGSRQALKVIFYLDSYHFSKLPSRLEGGASLRLHTALFTKVLENVEGLPSPGSQAAEDLQQDINAQSLEKVQQYYRKLRAFYLERSNLPTDASTTAVKIDQLIRPINALDELCRLMKSFVHPKPGAAGSVGAGLIPISSELCYRLGACQMVMCGTGMQRSTLSVSLEQAAILARSHGLLPKCIMQATDIMRKQGPRVEILAKNLRVKDQMPQGAPRLYRLCQPPVDGDL&sequence=DAQEARASQEASTED) | 1.841 |
| **Acidophilic serine/threonine kinase group (Acid_ST_kin)** | | | | |
| **Casein Kinase 1** | | | Gene Card [**CSNK1G2**](http://www.genecards.org/cgi-bin/carddisp.pl?gene=CSNK1G2) | |
| Site | Score | Percentile | Sequence | SA |
| S1115 | [0.3795](http://scansite.mit.edu/sitestats.phtml?site=S1115&position=1115&score=0.3795&percentile=0.200&zscore=-3.027&sequence=TEPTSGGSCDASLAE&protein=REX&motif=Casn_Kin1&source=histograms&db=_SWS_&class=VRT) | 0.200 % | [TEPTSGG**S**CDASLAE](http://scansite.mit.edu/showseq.phtml?prot_id=REX&motif=Casn_Kin1&protlength=1659&site=1115&protein=XMEAPSGSEPGGDGAGDCAHPDPRAPGAAAPSSGPGPCAAARESERQLRLRLCVLNEILGTERDYVGTLRFLQSAFLHRIRQNVADSVEKGLTEENVKVLFSNIEDILEVHKDFLAALEYCLHPEPQSQHELGNVFLKFKDKFCVYEEYCSNHEKALRLLVELNKIPTVRAFLLSCMLLGGRKTTDIPLEGYLLSPIQRICKYPLLLKELAKRTPGKHPDHPAVQSALQAMKTVCSNINETKRQMEKLEALEQLQSHIEGWEGSNLTDICTQLLLQGTLLKISAGNIQERAFFLFDNLLVYCKRKSRVTGSKKSTKRTKSINGSLYIFRGRINTEVMEVENVEDGTADYHSNGYTVTNGWKIHNTAKNKWFVCMAKTAEEKQKWLDAIIREREQRESLKLGMERDAYVMIAEKGEKLYHMMMNKKVNLIKDRRRKLSTVPKCFLGNEFVAWLLEIGEISKTEEGVNLGQALLENGIIHHVSDKHQFKNEQVMYRFRYDDGTYKARSELEDIMSKGVRLYCRLHSLYTPVIKDRDYHLKTYKSVLPGSKLVDWLLAQGDCQTREEAVALGVGLCNNGFMHHVLEKSEFRDESQYFRFHADEEMEGTSSKNKQLRNDFKLVENILAKRLLILPQEEDYGFDIEEKNKAVVVKSVQRGSLAEVAGLQVGRKIYSINEDLVFLRPFSEVESILNQSFCSRRPLRLLVATKAKEIIKIPDQPDTLCFQIRGAAPPYVYAVGRGSEAMAAGLCAGQCILKVNGSNVMNDGAPEVLEHFQAFRSRREEALGLYQWIYHTHEDAQEARASQEASTEDPSGEQAQEEDQADSAFPLLSLGPRLSLCEDSPMVTLTVDNVHLEHGVVYEYVSTAGVRCHVLEKIVEPRGCFGLTAKILEAFAANDSVFVENCRRLMALSSAIVTMPHFEFRNICDTKLESIGQRIACYQEFAAQLKSRVSPPFKQAPLEPHPLCGLDFCPTNCHINLMEVSYPKTTPSVGRSFSIRFGRKPSLIGLDPEQGHLNPMSYTQHCITTMAAPSWKCLPAAEGDPQGQGLHDGSFGPASGTLGQEDRGLSFLLKQEDREIQDAYLQLFTKLDVALKEMKQYVTQINRLLSTITEPTSGGSCDASLAEEASSLPLVSEESEMDRSDHGGIKKVCFKVAEEDQEDSGHDTMSYRDSYSECNSNRDSVLSYTSVRSNSSYLGSDEMGSGDELPCDMRIPSDKQDKLHGCLEHLFNQVDSINALLKGPVMSRAFEETKHFPMNHSLQEFKQKEECTIRGRSLIQISIQEDPWNLPNSIKTLVDNIQRYVEDGKNQLLLALLKCTDTELQLRRDAIFCQALVAAVCTFSKQLLAALGYRYNNNGEYEESSRDASRKWLEQVAATGVLLHCQSLLSPATVKEERTMLEDIWVTLSELDNVTFSFKQLDENYVANTNVFYHIEGSRQALKVIFYLDSYHFSKLPSRLEGGASLRLHTALFTKVLENVEGLPSPGSQAAEDLQQDINAQSLEKVQQYYRKLRAFYLERSNLPTDASTTAVKIDQLIRPINALDELCRLMKSFVHPKPGAAGSVGAGLIPISSELCYRLGACQMVMCGTGMQRSTLSVSLEQAAILARSHGLLPKCIMQATDIMRKQGPRVEILAKNLRVKDQMPQGAPRLYRLCQPPVDGDL&sequence=TEPTSGGSCDASLAE) | 0.272 |
| **Proline-dependent serine/threonine kinase group (Pro_ST_kin)** | | | | |
| **Cdk5 Kinase** | | | Gene Card [**CDK5**](http://www.genecards.org/cgi-bin/carddisp.pl?gene=CDK5) | |
| Site | Score | Percentile | Sequence | SA |
| T213 | [0.4009](http://scansite.mit.edu/sitestats.phtml?site=T213&position=213&score=0.4009&percentile=0.198&zscore=-4.154&sequence=LKELAKRTPGKHPDH&protein=REX&motif=Cdk5_Kin&source=histograms&db=_SWS_&class=VRT) | 0.198 % | [LKELAKR**T**PGKHPDH](http://scansite.mit.edu/showseq.phtml?prot_id=REX&motif=Cdk5_Kin&protlength=1659&site=213&protein=XMEAPSGSEPGGDGAGDCAHPDPRAPGAAAPSSGPGPCAAARESERQLRLRLCVLNEILGTERDYVGTLRFLQSAFLHRIRQNVADSVEKGLTEENVKVLFSNIEDILEVHKDFLAALEYCLHPEPQSQHELGNVFLKFKDKFCVYEEYCSNHEKALRLLVELNKIPTVRAFLLSCMLLGGRKTTDIPLEGYLLSPIQRICKYPLLLKELAKRTPGKHPDHPAVQSALQAMKTVCSNINETKRQMEKLEALEQLQSHIEGWEGSNLTDICTQLLLQGTLLKISAGNIQERAFFLFDNLLVYCKRKSRVTGSKKSTKRTKSINGSLYIFRGRINTEVMEVENVEDGTADYHSNGYTVTNGWKIHNTAKNKWFVCMAKTAEEKQKWLDAIIREREQRESLKLGMERDAYVMIAEKGEKLYHMMMNKKVNLIKDRRRKLSTVPKCFLGNEFVAWLLEIGEISKTEEGVNLGQALLENGIIHHVSDKHQFKNEQVMYRFRYDDGTYKARSELEDIMSKGVRLYCRLHSLYTPVIKDRDYHLKTYKSVLPGSKLVDWLLAQGDCQTREEAVALGVGLCNNGFMHHVLEKSEFRDESQYFRFHADEEMEGTSSKNKQLRNDFKLVENILAKRLLILPQEEDYGFDIEEKNKAVVVKSVQRGSLAEVAGLQVGRKIYSINEDLVFLRPFSEVESILNQSFCSRRPLRLLVATKAKEIIKIPDQPDTLCFQIRGAAPPYVYAVGRGSEAMAAGLCAGQCILKVNGSNVMNDGAPEVLEHFQAFRSRREEALGLYQWIYHTHEDAQEARASQEASTEDPSGEQAQEEDQADSAFPLLSLGPRLSLCEDSPMVTLTVDNVHLEHGVVYEYVSTAGVRCHVLEKIVEPRGCFGLTAKILEAFAANDSVFVENCRRLMALSSAIVTMPHFEFRNICDTKLESIGQRIACYQEFAAQLKSRVSPPFKQAPLEPHPLCGLDFCPTNCHINLMEVSYPKTTPSVGRSFSIRFGRKPSLIGLDPEQGHLNPMSYTQHCITTMAAPSWKCLPAAEGDPQGQGLHDGSFGPASGTLGQEDRGLSFLLKQEDREIQDAYLQLFTKLDVALKEMKQYVTQINRLLSTITEPTSGGSCDASLAEEASSLPLVSEESEMDRSDHGGIKKVCFKVAEEDQEDSGHDTMSYRDSYSECNSNRDSVLSYTSVRSNSSYLGSDEMGSGDELPCDMRIPSDKQDKLHGCLEHLFNQVDSINALLKGPVMSRAFEETKHFPMNHSLQEFKQKEECTIRGRSLIQISIQEDPWNLPNSIKTLVDNIQRYVEDGKNQLLLALLKCTDTELQLRRDAIFCQALVAAVCTFSKQLLAALGYRYNNNGEYEESSRDASRKWLEQVAATGVLLHCQSLLSPATVKEERTMLEDIWVTLSELDNVTFSFKQLDENYVANTNVFYHIEGSRQALKVIFYLDSYHFSKLPSRLEGGASLRLHTALFTKVLENVEGLPSPGSQAAEDLQQDINAQSLEKVQQYYRKLRAFYLERSNLPTDASTTAVKIDQLIRPINALDELCRLMKSFVHPKPGAAGSVGAGLIPISSELCYRLGACQMVMCGTGMQRSTLSVSLEQAAILARSHGLLPKCIMQATDIMRKQGPRVEILAKNLRVKDQMPQGAPRLYRLCQPPVDGDL&sequence=LKELAKRTPGKHPDH) | 3.964 |
| **Kinase binding site group (Kin_bind)** | | | | |
| **Erk D-domain** | | | Gene Card [**MAPK1**](http://www.genecards.org/cgi-bin/carddisp.pl?gene=MAPK1) | |
| Site | Score | Percentile | Sequence | SA |
| V702 | [0.5718](http://scansite.mit.edu/sitestats.phtml?site=V702&position=702&score=0.5718&percentile=0.160&zscore=-3.421&sequence=RRPLRLLVATKAKEI&protein=REX&motif=ErkDD&source=histograms&db=_SWS_&class=VRT) | 0.160 % | [RRPLRLL**V**ATKAKEI](http://scansite.mit.edu/showseq.phtml?prot_id=REX&motif=ErkDD&protlength=1659&site=702&protein=XMEAPSGSEPGGDGAGDCAHPDPRAPGAAAPSSGPGPCAAARESERQLRLRLCVLNEILGTERDYVGTLRFLQSAFLHRIRQNVADSVEKGLTEENVKVLFSNIEDILEVHKDFLAALEYCLHPEPQSQHELGNVFLKFKDKFCVYEEYCSNHEKALRLLVELNKIPTVRAFLLSCMLLGGRKTTDIPLEGYLLSPIQRICKYPLLLKELAKRTPGKHPDHPAVQSALQAMKTVCSNINETKRQMEKLEALEQLQSHIEGWEGSNLTDICTQLLLQGTLLKISAGNIQERAFFLFDNLLVYCKRKSRVTGSKKSTKRTKSINGSLYIFRGRINTEVMEVENVEDGTADYHSNGYTVTNGWKIHNTAKNKWFVCMAKTAEEKQKWLDAIIREREQRESLKLGMERDAYVMIAEKGEKLYHMMMNKKVNLIKDRRRKLSTVPKCFLGNEFVAWLLEIGEISKTEEGVNLGQALLENGIIHHVSDKHQFKNEQVMYRFRYDDGTYKARSELEDIMSKGVRLYCRLHSLYTPVIKDRDYHLKTYKSVLPGSKLVDWLLAQGDCQTREEAVALGVGLCNNGFMHHVLEKSEFRDESQYFRFHADEEMEGTSSKNKQLRNDFKLVENILAKRLLILPQEEDYGFDIEEKNKAVVVKSVQRGSLAEVAGLQVGRKIYSINEDLVFLRPFSEVESILNQSFCSRRPLRLLVATKAKEIIKIPDQPDTLCFQIRGAAPPYVYAVGRGSEAMAAGLCAGQCILKVNGSNVMNDGAPEVLEHFQAFRSRREEALGLYQWIYHTHEDAQEARASQEASTEDPSGEQAQEEDQADSAFPLLSLGPRLSLCEDSPMVTLTVDNVHLEHGVVYEYVSTAGVRCHVLEKIVEPRGCFGLTAKILEAFAANDSVFVENCRRLMALSSAIVTMPHFEFRNICDTKLESIGQRIACYQEFAAQLKSRVSPPFKQAPLEPHPLCGLDFCPTNCHINLMEVSYPKTTPSVGRSFSIRFGRKPSLIGLDPEQGHLNPMSYTQHCITTMAAPSWKCLPAAEGDPQGQGLHDGSFGPASGTLGQEDRGLSFLLKQEDREIQDAYLQLFTKLDVALKEMKQYVTQINRLLSTITEPTSGGSCDASLAEEASSLPLVSEESEMDRSDHGGIKKVCFKVAEEDQEDSGHDTMSYRDSYSECNSNRDSVLSYTSVRSNSSYLGSDEMGSGDELPCDMRIPSDKQDKLHGCLEHLFNQVDSINALLKGPVMSRAFEETKHFPMNHSLQEFKQKEECTIRGRSLIQISIQEDPWNLPNSIKTLVDNIQRYVEDGKNQLLLALLKCTDTELQLRRDAIFCQALVAAVCTFSKQLLAALGYRYNNNGEYEESSRDASRKWLEQVAATGVLLHCQSLLSPATVKEERTMLEDIWVTLSELDNVTFSFKQLDENYVANTNVFYHIEGSRQALKVIFYLDSYHFSKLPSRLEGGASLRLHTALFTKVLENVEGLPSPGSQAAEDLQQDINAQSLEKVQQYYRKLRAFYLERSNLPTDASTTAVKIDQLIRPINALDELCRLMKSFVHPKPGAAGSVGAGLIPISSELCYRLGACQMVMCGTGMQRSTLSVSLEQAAILARSHGLLPKCIMQATDIMRKQGPRVEILAKNLRVKDQMPQGAPRLYRLCQPPVDGDL&sequence=RRPLRLLVATKAKEI) | 0.337 |
| **Erk D-domain** | | | Gene Card [**MAPK1**](http://www.genecards.org/cgi-bin/carddisp.pl?gene=MAPK1) | |
| Site | Score | Percentile | Sequence | SA |
| L1005 | [0.4441](http://scansite.mit.edu/sitestats.phtml?site=L1005&position=1005&score=0.4441&percentile=0.012&zscore=-4.309&sequence=RKPSLIGLDPEQGHL&protein=REX&motif=ErkDD&source=histograms&db=_SWS_&class=VRT) | 0.012 % | [RKPSLIG**L**DPEQGHL](http://scansite.mit.edu/showseq.phtml?prot_id=REX&motif=ErkDD&protlength=1659&site=1005&protein=XMEAPSGSEPGGDGAGDCAHPDPRAPGAAAPSSGPGPCAAARESERQLRLRLCVLNEILGTERDYVGTLRFLQSAFLHRIRQNVADSVEKGLTEENVKVLFSNIEDILEVHKDFLAALEYCLHPEPQSQHELGNVFLKFKDKFCVYEEYCSNHEKALRLLVELNKIPTVRAFLLSCMLLGGRKTTDIPLEGYLLSPIQRICKYPLLLKELAKRTPGKHPDHPAVQSALQAMKTVCSNINETKRQMEKLEALEQLQSHIEGWEGSNLTDICTQLLLQGTLLKISAGNIQERAFFLFDNLLVYCKRKSRVTGSKKSTKRTKSINGSLYIFRGRINTEVMEVENVEDGTADYHSNGYTVTNGWKIHNTAKNKWFVCMAKTAEEKQKWLDAIIREREQRESLKLGMERDAYVMIAEKGEKLYHMMMNKKVNLIKDRRRKLSTVPKCFLGNEFVAWLLEIGEISKTEEGVNLGQALLENGIIHHVSDKHQFKNEQVMYRFRYDDGTYKARSELEDIMSKGVRLYCRLHSLYTPVIKDRDYHLKTYKSVLPGSKLVDWLLAQGDCQTREEAVALGVGLCNNGFMHHVLEKSEFRDESQYFRFHADEEMEGTSSKNKQLRNDFKLVENILAKRLLILPQEEDYGFDIEEKNKAVVVKSVQRGSLAEVAGLQVGRKIYSINEDLVFLRPFSEVESILNQSFCSRRPLRLLVATKAKEIIKIPDQPDTLCFQIRGAAPPYVYAVGRGSEAMAAGLCAGQCILKVNGSNVMNDGAPEVLEHFQAFRSRREEALGLYQWIYHTHEDAQEARASQEASTEDPSGEQAQEEDQADSAFPLLSLGPRLSLCEDSPMVTLTVDNVHLEHGVVYEYVSTAGVRCHVLEKIVEPRGCFGLTAKILEAFAANDSVFVENCRRLMALSSAIVTMPHFEFRNICDTKLESIGQRIACYQEFAAQLKSRVSPPFKQAPLEPHPLCGLDFCPTNCHINLMEVSYPKTTPSVGRSFSIRFGRKPSLIGLDPEQGHLNPMSYTQHCITTMAAPSWKCLPAAEGDPQGQGLHDGSFGPASGTLGQEDRGLSFLLKQEDREIQDAYLQLFTKLDVALKEMKQYVTQINRLLSTITEPTSGGSCDASLAEEASSLPLVSEESEMDRSDHGGIKKVCFKVAEEDQEDSGHDTMSYRDSYSECNSNRDSVLSYTSVRSNSSYLGSDEMGSGDELPCDMRIPSDKQDKLHGCLEHLFNQVDSINALLKGPVMSRAFEETKHFPMNHSLQEFKQKEECTIRGRSLIQISIQEDPWNLPNSIKTLVDNIQRYVEDGKNQLLLALLKCTDTELQLRRDAIFCQALVAAVCTFSKQLLAALGYRYNNNGEYEESSRDASRKWLEQVAATGVLLHCQSLLSPATVKEERTMLEDIWVTLSELDNVTFSFKQLDENYVANTNVFYHIEGSRQALKVIFYLDSYHFSKLPSRLEGGASLRLHTALFTKVLENVEGLPSPGSQAAEDLQQDINAQSLEKVQQYYRKLRAFYLERSNLPTDASTTAVKIDQLIRPINALDELCRLMKSFVHPKPGAAGSVGAGLIPISSELCYRLGACQMVMCGTGMQRSTLSVSLEQAAILARSHGLLPKCIMQATDIMRKQGPRVEILAKNLRVKDQMPQGAPRLYRLCQPPVDGDL&sequence=RKPSLIGLDPEQGHL) | 0.586 |
| **Lipid binding group (Lip_bind)** | | | | |
| **PIP3-binding PH** | | | Gene Card [**PIP3-E**](http://www.genecards.org/cgi-bin/carddisp.pl?gene=PIP3-E) | |
| Site | Score | Percentile | Sequence | SA |
| F138 | [0.6024](http://scansite.mit.edu/sitestats.phtml?site=F138&position=138&score=0.6024&percentile=0.103&zscore=-3.603&sequence=LGNVFLKFKDKFCVY&protein=REX&motif=PIP3_PH&source=histograms&db=_SWS_&class=VRT) | 0.103 % | [LGNVFLK**F**KDKFCVY](http://scansite.mit.edu/showseq.phtml?prot_id=REX&motif=PIP3_PH&protlength=1659&site=138&protein=XMEAPSGSEPGGDGAGDCAHPDPRAPGAAAPSSGPGPCAAARESERQLRLRLCVLNEILGTERDYVGTLRFLQSAFLHRIRQNVADSVEKGLTEENVKVLFSNIEDILEVHKDFLAALEYCLHPEPQSQHELGNVFLKFKDKFCVYEEYCSNHEKALRLLVELNKIPTVRAFLLSCMLLGGRKTTDIPLEGYLLSPIQRICKYPLLLKELAKRTPGKHPDHPAVQSALQAMKTVCSNINETKRQMEKLEALEQLQSHIEGWEGSNLTDICTQLLLQGTLLKISAGNIQERAFFLFDNLLVYCKRKSRVTGSKKSTKRTKSINGSLYIFRGRINTEVMEVENVEDGTADYHSNGYTVTNGWKIHNTAKNKWFVCMAKTAEEKQKWLDAIIREREQRESLKLGMERDAYVMIAEKGEKLYHMMMNKKVNLIKDRRRKLSTVPKCFLGNEFVAWLLEIGEISKTEEGVNLGQALLENGIIHHVSDKHQFKNEQVMYRFRYDDGTYKARSELEDIMSKGVRLYCRLHSLYTPVIKDRDYHLKTYKSVLPGSKLVDWLLAQGDCQTREEAVALGVGLCNNGFMHHVLEKSEFRDESQYFRFHADEEMEGTSSKNKQLRNDFKLVENILAKRLLILPQEEDYGFDIEEKNKAVVVKSVQRGSLAEVAGLQVGRKIYSINEDLVFLRPFSEVESILNQSFCSRRPLRLLVATKAKEIIKIPDQPDTLCFQIRGAAPPYVYAVGRGSEAMAAGLCAGQCILKVNGSNVMNDGAPEVLEHFQAFRSRREEALGLYQWIYHTHEDAQEARASQEASTEDPSGEQAQEEDQADSAFPLLSLGPRLSLCEDSPMVTLTVDNVHLEHGVVYEYVSTAGVRCHVLEKIVEPRGCFGLTAKILEAFAANDSVFVENCRRLMALSSAIVTMPHFEFRNICDTKLESIGQRIACYQEFAAQLKSRVSPPFKQAPLEPHPLCGLDFCPTNCHINLMEVSYPKTTPSVGRSFSIRFGRKPSLIGLDPEQGHLNPMSYTQHCITTMAAPSWKCLPAAEGDPQGQGLHDGSFGPASGTLGQEDRGLSFLLKQEDREIQDAYLQLFTKLDVALKEMKQYVTQINRLLSTITEPTSGGSCDASLAEEASSLPLVSEESEMDRSDHGGIKKVCFKVAEEDQEDSGHDTMSYRDSYSECNSNRDSVLSYTSVRSNSSYLGSDEMGSGDELPCDMRIPSDKQDKLHGCLEHLFNQVDSINALLKGPVMSRAFEETKHFPMNHSLQEFKQKEECTIRGRSLIQISIQEDPWNLPNSIKTLVDNIQRYVEDGKNQLLLALLKCTDTELQLRRDAIFCQALVAAVCTFSKQLLAALGYRYNNNGEYEESSRDASRKWLEQVAATGVLLHCQSLLSPATVKEERTMLEDIWVTLSELDNVTFSFKQLDENYVANTNVFYHIEGSRQALKVIFYLDSYHFSKLPSRLEGGASLRLHTALFTKVLENVEGLPSPGSQAAEDLQQDINAQSLEKVQQYYRKLRAFYLERSNLPTDASTTAVKIDQLIRPINALDELCRLMKSFVHPKPGAAGSVGAGLIPISSELCYRLGACQMVMCGTGMQRSTLSVSLEQAAILARSHGLLPKCIMQATDIMRKQGPRVEILAKNLRVKDQMPQGAPRLYRLCQPPVDGDL&sequence=LGNVFLKFKDKFCVY) | 2.186 |
